# Effectiveness of Choosing Wisely recommendations in reducing physiotherapists’ intentions to refer for imaging and use electrotherapy for low back pain: a randomised controlled experiment

**DOI:** 10.1136/bmjopen-2024-097202

**Published:** 2025-06-26

**Authors:** Priti Kharel, Chris G Maher, Andrew R Gamble, Giovanni E Ferreira, Joshua R Zadro

**Affiliations:** 1 Sydney School of Public Health, Faculty of Medicine and Health, The University of Sydney, Sydney, New South Wales, Australia; 2 Institute for Musculoskeletal Health, Sydney Local Health District, Sydney, New South Wales, Australia; 3 Sydney Musculoskeletal Health, Sydney School of Public Health, Faculty of Medicine and Health, The University of Sydney, Sydney, New South Wales, Australia

**Keywords:** Physical Therapy Modalities, Back pain, Diagnostic Imaging

## Abstract

**Abstract:**

**Question:**

What is the effect of Choosing Wisely recommendations on physiotherapists’ intentions to refer for imaging and use electrotherapy for low back pain?

**Design:**

Three-arm parallel-group online randomised controlled trial.

**Participants:**

Physiotherapists who treat people with low back pain.

**Intervention:**

Participants were randomised to receive: (a) two *original* Australian Physiotherapy Association Choosing Wisely recommendations about low back pain*,* (b) two *optimised* versions of these recommendations based on previous research and (c) *no recommendations*. Participants were then directed to read three clinical vignettes of a person with low back pain and respond to questions regarding each vignette.

**Outcome measures:**

Primary outcomes were physiotherapists’ intentions to refer for imaging and use electrotherapy for low back pain. Secondary outcomes were physiotherapists’ intentions to use other treatments for low back pain, the influence of the recommendations on decision-making in the vignettes and familiarity with the recommendations.

**Results:**

723 participants opened the survey and 473 (65%) provided complete responses. Across all vignettes, there were no statistically significant differences in intentions to refer for imaging or use electrotherapy between those who received *Choosing Wisely recommendations* versus *no recommendation* (imaging ORs ranging from 0.7 (95% CI 0.5 to 1.0) to 0.9 (0.6 to 1.4); electrotherapy ORs ranging from 0.9 (0.5 to 1.7) to 1.1 (0.7 to 2.0)). Similarly, no significant differences were observed between those who received *optimised* versus *original recommendations* for all three vignettes.

**Conclusion:**

Our study suggests simply presenting Choosing Wisely recommendations to physiotherapists does not influence their intentions to refer for imaging or use electrotherapy for low back pain, even if the language of the recommendations is optimised.

**STRENGTHS AND LIMITATIONS OF THIS STUDY**

- The study’s randomised design allowed for an accurate assessment of the recommendations’ effects on physiotherapists’ intentions to refer for imaging and use electrotherapy.
- Participants were not excluded based on age, gender, clinical experience or specialisation, capturing a diverse range of responses.
- Low dropout rates after randomisation reduced the risk of attrition bias.
- The online scenario-based design may not fully reflect physiotherapists’ decision-making during real-world consultations.
- The test and treatment options in the vignette were not randomised, which may have led to more participants selecting the first few options listed.

## Introduction

 Low back pain (LBP) is the most common musculoskeletal condition worldwide^1^ and in Australia.^2^ LBP imposes a financial burden of $A4.8 billion on the Australian healthcare system each year,^3^ and it is the leading cause of both reduced work productivity and early retirement.^4 5^ In 2019, LBP was responsible for 44% of the disability burden of all musculoskeletal conditions in Australia.^6^ To exacerbate the situation, a significant number of individuals suffering from LBP receive inappropriate care, including both underuse of recommended care and overuse of non-recommended care.^7^

Choosing Wisely is a global campaign that has been encouraging healthcare professionals, such as physiotherapists, to deliver high-quality patient care since 2012.^8^ Currently, more than 250 professional societies across over 20 countries have joined the campaign and have released their list of recommendations targeting inappropriate care in their profession.^8^ This includes the Australian Physiotherapy Association (APA), which has released six recommendations targeting inappropriate tests and treatments relevant to physiotherapists. Two of these recommendations are relevant to the management of LBP: ‘Don’t request imaging for patients with non-specific low back pain and no indicators of a serious cause for low back pain’ and ‘Avoid using electrotherapy modalities in the management of patients with low back pain’.

Numerous studies across various health disciplines have investigated the effect of the Choosing Wisely campaign on reducing the overuse of healthcare.^9^,^11^ However, findings have been mixed and no high-quality studies have evaluated the effect of the Choosing Wisely recommendations on physiotherapy practice. A recent study investigating physiotherapists’ attitudes, views and beliefs about the APA Choosing Wisely recommendations found that the language of the recommendations was a key barrier to physiotherapists adopting them and making simple modifications to the language could solve this issue.^12^ Building on this, a Best-Worst Scaling survey with 215 physiotherapists found that physiotherapists were more willing to follow Choosing Wisely recommendations that were more detailed, positively framed and provided alternatives to low-value care.^12^ Together, these studies indicate that there is room to improve the wording of the existing APA Choosing Wisely recommendations.

Overall, there is a need to assess the impact of the Choosing Wisely recommendations on reducing physiotherapists’ use of low-value care and investigate whether optimising the language of the recommendations can enhance their impact. The primary aim of this study was to investigate the effect of (a) the original Choosing Wisely recommendations and (b) optimised Choosing Wisely recommendations on physiotherapists’ intentions to refer for imaging and use electrotherapy for LBP compared with *no recommendation*. The optimised Choosing Wisely recommendations in our previous study were modified to be positively framed, detailed and provide an alternative to low-value care.^13^ The secondary aims were to investigate the effect of the recommendations on physiotherapists’ intentions to use other treatments in the management of people with LBP, explore the influence of the recommendations on physiotherapists’ decision-making in the vignettes, and describe physiotherapists’ familiarity and agreement with the Choosing Wisely recommendations.

Therefore, the study question for this randomised experiment was:

What is the effect of Choosing Wisely recommendations on physiotherapists’ intentions to refer for imaging and use electrotherapy for LBP?

## Methods

### Design

We conducted an online three-arm parallel-group randomised controlled trial, reported as per the Consolidated Standards of Reporting Trials statement (online supplemental appendix 1).^14^ We pilot tested our online survey with eight researchers prior to recruitment.

### Participant selection and recruitment

We recruited registered and practising physiotherapists who had treated patients with LBP in the past 12 months. We did not restrict participants based on their age (provided they were 18 years or older), sex, clinical experience, area of specialty or country of practice. We recruited participants via social media (eg, Facebook, X-formerly Twitter) and through the market research company, Qualtrics. Qualtrics recruits eligible participants from over 20 panel providers globally. Most participants come from traditional, actively managed market research panels, although social media is occasionally used for recruitment purposes. Qualtrics uses random sampling methods and compensates participants by offering incentives such as cash, airline miles or gift cards, uses advanced digital fingerprinting technology and verifies each IP address to ensure the validity of responses and prevent duplication.^15^

Participants clicking on the link to the study were provided a brief explanation of the study (online supplemental appendix 2) and were directed to read the Participant Information Statement (online supplemental appendix 3). Participants then read a Participant Consent Form and indicated their consent to participate by checking a box before proceeding to the survey (online supplemental appendix 4). The study objectives were undisclosed from the participants recruited from the market research panels to ensure participants did not pretend to be physiotherapists to receive an incentive through Qualtrics (online supplemental appendix 5).

### Data collection

Consenting and eligible participants were asked to provide the following demographic and professional information: gender, age, physiotherapy qualification, country of practice, clinical area of interest, clinical setting(s), whether the participant is involved in research and/or teaching or other professional activities, years practising as a physiotherapist, knowledge of imaging guidelines and evidence on the effectiveness of electrotherapy for LBP, and their influence on practice. Following this, participants were randomised into three groups. The survey was open from November 2023 to March 2024.

### Intervention

**Figure 1:**
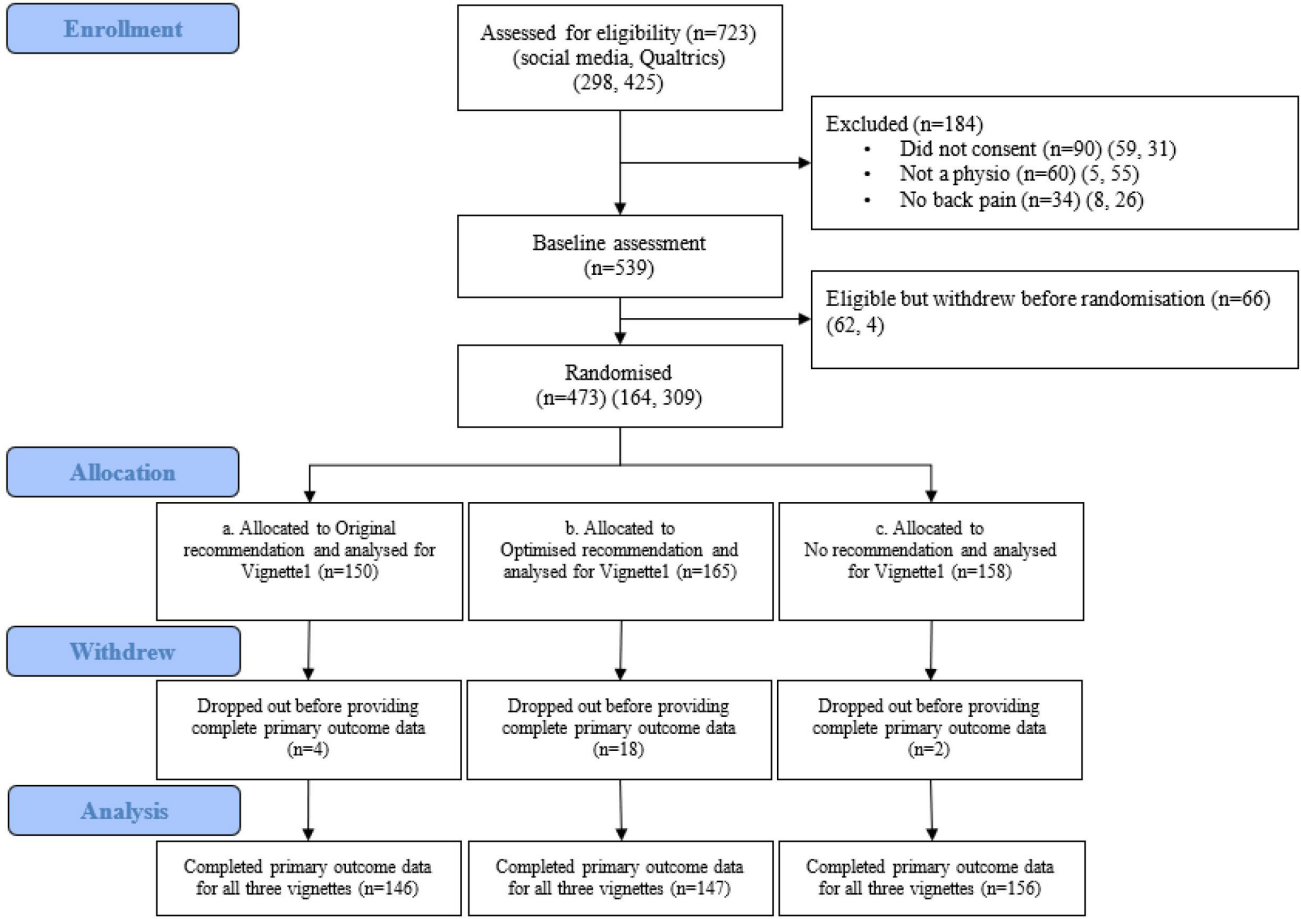
Flow of participants through the study.

All participants (recruited via social media or Qualtrics panel) were randomised (1:1:1 ratio) to one of the three groups (figure 1) through a randomisation process facilitated in the Qualtrics survey software:

1. *Original Choosing Wisely recommendation:* participants were shown a short description of the Choosing Wisely campaign and the two original APA recommendations related to LBP.
2. *Optimised Choosing Wisely recommendations:* participants were shown a short description of the Choosing Wisely campaign and the two optimised APA recommendations related to LBP. The two optimised recommendations were previously modified to be positive, more detailed than the original and offer alternatives to low-value care.^13^ Physiotherapists in our previous study were 13% more willing to follow recommendations with those characteristics compared with those without them.^13^ Table 1 outlines the original and optimised recommendations.
3. *No recommendation*: participants did not receive any recommendations or information about the Choosing Wisely campaign (figure 1).

**Table 1:** Original and optimised Choosing Wisely recommendations

Original Choosing Wisely recommendations	Optimised Choosing Wisely recommendations
Do not request imaging for patients with non-specific low back pain and no indicators of a serious cause for low back pain Avoid using electrotherapy modalities in the management of patients with low back pain	Physiotherapists should not request imaging for patients with non-specific low back pain and no indicators of a serious cause for low back pain as the findings are unlikely to positively guide management. Physiotherapists should instead explain why imaging is not required Physiotherapists should not use electrotherapy modalities in the management of patients with low back pain as they are not superior to placebo. Physiotherapists should instead give advice to stay active and reassurance

### Hypothetical vignettes

Participants were then directed to read three clinical vignettes and respond to questions regarding each vignette. The vignettes used in this study were adapted from a study investigating LBP guideline adherence among Brazilian physiotherapists.^16^

All three vignettes provided short patient histories (including signs and symptoms), described the physical examination findings and varying clinical presentation. Imaging was deemed inappropriate for vignettes 1 (acute LBP with no serious indicators) and 2 (insidious LBP with no serious indicators), but appropriate for vignette 3 (sudden-onset low back pain and clear indicators of a potential serious pathology), while electrotherapy was considered inappropriate for all three vignettes^17^ (online supplemental appendix 5). Participants were asked to read the three vignettes and imagine that they were the physiotherapist treating the patient. After reading the vignette, participants were asked what tests and treatments they would provide to the patients in the vignettes—based on a list of pre-specified options, which included diagnostic imaging, electrotherapy and various other commonly provided physiotherapy treatments (online supplemental appendix 5). Participants were allowed to select as many options as they liked and were asked to consider options that may be applicable beyond the first session.

### Outcome measures

Outcomes were collected immediately after participants read each vignette. Our outcome measures are listed below with their variable categories, scale and response options (table 2).

**Table 2:** Outcome measures

Outcome measures	Variable categories	Response scale	Response options*
Primary outcomes			
Intentions to refer for imaging	Categorical	Multiple choice	Refer for bone scan Refer for CT scan Refer for MRI Refer for radiographs Refer for ultrasound
Intentions to use electrotherapy	Categorical	Multiple choice	Interferential current or transcutaneous electrical nerve stimulation (TENS) Laser or ultrasound
Secondary outcomes			
Intentions to provide other treatments	Categorical	Multiple choice	Refer to doctor Refer to the medical doctor and maintain physiotherapy treatment Refer to the medical doctor without intervention Advice and education Advice to maintain an upright posture during bending and lifting Advice to pursue or maintain an active lifestyle Pain neuroscience education (eg, ‘pain does not equal damage’) Psychologically informed physiotherapy (behavioural therapy in addition to usual care physiotherapy) Exercise Aerobic and fitness exercises Back school (a booklet that includes instructions on a home exercise programme) Directional preference exercises: extension Directional preference exercises: flexion Gradual exercise exposure Lumbar stabilisation exercises Heat, cold and bracing Ice or heat Lumbar brace or corset Bed rest Manual therapy Mechanical traction Neurodynamic mobilisation Spinal manipulation (thrust) Spinal mobilisation (non-thrust) Other (eg, cupping, massage and taping, relaxation, soft tissue mobilisation, explore medication efficiency)
Influence of Choosing Wisely recommendations on decision-making in the vignettes	Ordinal	5-point Likert	A great deal A fair amount Somewhat Slightly Not at all
Familiarity with Choosing Wisely recommendations	Ordinal	5-point Likert	Extremely familiar Very familiar Moderately familiar Slightly familiar Not familiar at all
Agreement with Choosing Wisely recommendations against imaging for low back pain	Ordinal	5-point Likert	Strongly agree Somewhat agree Neither agree nor disagree Somewhat disagree Strongly disagree
Agreement with Choosing Wisely recommendations against electrotherapy for low back pain	Ordinal	5-point Likert	Strongly agree Somewhat agree Neither agree nor disagree Somewhat disagree Strongly disagree

* All imaging and treatment options were included in the same question following each vignette.

### Sample size calculation

We calculated that a sample size of 468 (156 per group) would provide 80% power to detect a 10% between-group difference for our primary comparison (*Choosing Wisely recommendations* vs *no recommendation*) in the percentage of physiotherapists intending to refer for imaging or use electrotherapy for LBP (ie, 15% of physiotherapists intending to refer for imaging and use electrotherapy in the intervention groups and 25% in the control group). This assumes a two-tailed alpha of 0.05 and a 15% dropout. A 10% between-group effect for our primary outcomes was selected as it is an effect size commonly used for studies investigating treatment choices for LBP.^18 19^ The base rate of 25% of physiotherapists referring for imaging and using electrotherapy for LBP was taken from a systematic review investigating what treatments physiotherapists provide for musculoskeletal conditions^20^ and a survey investigating physiotherapists’ imaging referral rates for low back pain.^21^ We recruited slightly more participants than required (N=473).

### Data analysis

Data analyses were performed according to an intention-to-treat approach. We used descriptive statistics to summarise participants’ demographic and professional characteristics. Multivariable logistic regression models were used to examine the effect of Choosing Wisely recommendations on physiotherapists’ intentions to refer for imaging or use electrotherapy for LBP in each vignette. The regression included a comparison of Choosing Wisely recommendations (both *optimised* and *original recommendations* combined) versus *no recommendation*, followed by a comparison of *optimised* versus *original Choosing Wisely recommendations*. The five imaging tests (bone scan, CT scan, MRI, radiographs and ultrasound) that physiotherapists could refer to were collapsed into one imaging variable for analysis. The four electrotherapy treatment options (interferential, transcutaneous electrical nerve stimulation, laser and ultrasound) were collapsed into one electrotherapy variable for analysis. To explore the influence of poor-quality responses on our main findings, we conducted a sensitivity analysis where we excluded participants with (a) irrelevant answers to open-ended questions, (b) repeated phrases across different free-text fields from the same participant, (c) identical responses in the same free text field to another participant (ie, duplicate response) and (d) a survey completion time of <10 min. Analyses were adjusted for participants’ involvement in research or teaching (for all analyses) and participants’ being ‘a fair amount’ (or more) influenced by imaging guidelines for the imaging analyses, and ‘a fair amount’ (or more) influenced by evidence on the effectiveness of electrotherapy for the electrotherapy analyses.

To explore physiotherapists’ intentions to provide other treatments in the management of people with LBP, we collapsed related tests and treatment options into broad categories as outlined in online supplemental appendix 6. We used descriptive statistics to summarise physiotherapists’ familiarity and agreement with the recommendations and their influence on the decision-making in the vignettes (only for the two groups which received the recommendations). All analyses were conducted in Stata SE V.18.0 and were reported as adjusted ORs and 95% CIs.

### Patient and public involvement

We did not involve patients and members of the public in the design of this study.

## Results

### Flow of participants through the study

Of the 723 participants who opened the survey, 184 were excluded (figure 1) and 66 withdrew before randomisation. This left 473 participants who were randomised to the three groups. Dropout rates were 2.7% for participants randomised to the *Original recommendations*, 10.9% for the *Optimised recommendations group* and 1.3% for the *no recommendation group*. The demographic characteristics of participants who exited the survey before responding to the first vignette were similar to those who completed the survey (online supplemental appendix 7). The median (IQR) survey duration for those randomised was 14.6 min (9–22).

### Participant characteristics

Most participants were 30–39 (n=171, 36%) or 40–49 years old (n=170, 36%), and 193 (41%) were female. 44% had >10 years of clinical experience (n=208) and 56% worked in a private setting (n=328). About one-third of participants had a master’s degree (n=228, 32%), practised in the USA (n=164, 35%), and worked as a musculoskeletal physiotherapist (n=398, 31%) (table 3).

**Table 3:** Characteristics of participants

Characteristics (N=473)	Original recommendation (n=150)	Optimised recommendation (n=165)	No recommendation (n=158)	Total (N=473)
Age (years), n (%)				
<20	0 (0%)	1 (1%)	1 (1%)	2 (1%)
20–29	25 (17%)	26 (16%)	26 (17%)	77 (16%)
30–39	46 (31%)	66 (40%)	59 (37%)	171 (36%)
40–49	57 (38%)	59 (36%)	54 (34%)	170 (36%)
50+	22 (14%)	13 (8%)	18 (11%)	53 (11%)
Sex, n (%)				
Male	85 (57%)	102 (62%)	93 (59%)	280 (59%)
Female	65 (43%)	63 (38%)	65 (41%)	193 (41%)
Number of LBP patients seen in last 12 months, n (%)				
1–5	4 (3%)	10 (6%)	8 (5%)	22 (5%)
6–50	62 (41%)	66 (40%)	75 (48%)	203 (43%)
51–100	53 (35%)	56 (34%)	49 (31%)	158 (33%)
>100	31 (21%)	33 (20%)	26 (16%)	90 (19%)
Qualification*, n (%)				
Bachelors	66 (44%)	67 (41%)	77 (49%)	210 (44%)
Masters	83 (55%)	70 (42%)	75 (47%)	228 (48%)
PhD	21 (14%)	15 (9%)	19 (12%)	55 (12%)
Titling	12 (8%)	11 (7%)	6 (4%)	29 (6%)
Specialisation	53 (35%)	64 (39%)	55 (35%)	172 (36%)
Other qualification	5 (3%)	7 (4%)	7 (4%)	19 (4%)
Country of practice, n (%)				
USA	47 (31%)	70 (42%)	47 (30%)	164 (31%)
UK	43 (29%)	47 (28%)	48 (30%)	138 (29%)
Australia	36 (24%)	22 (13%)	35 (22%)	93 (24%)
Others	24 (16%)	26 (16%)	28 (18%)	78 (16%)
Years of experience, n (%)				
<1 year	5 (3%)	5 (3%)	7 (5%)	17 (4%)
1–5 years	25 (17%)	35 (21%)	32 (20%)	92 (20%)
6–10 years	53 (35%)	51 (31%)	52 (33%)	156 (33%)
10+	67 (45%)	74 (45%)	67 (42%)	208 (44%)
Clinical area of interest*, n (%)				
Musculoskeletal	130 (87%)	130 (79%)	138 (87%)	398 (84%)
Sports	63 (42%)	79 (48%)	66 (42%)	208 (44%)
Orthopaedics	59 (39%)	66 (15%)	57 (36%)	182 (38%)
Cardiothoracic	49 (33%)	40 (24%)	48 (30%)	137 (29%)
Neurology	44 (29%)	40 (24%)	48 (30%)	132 (28%)
Ergonomics and occupational health	14 (9%)	25 (15%)	24 (15%)	63 (13%)
Continence and women’s health	13 (9%)	19 (12%)	13 (8%)	45 (10%)
Gerontology	15 (10%)	14 (8%)	15 (9%)	44 (9%)
Paediatrics	11 (7%)	11 (7%)	13 (8%)	35 (7%)
Oncology	11 (7%)	6 (4%)	11 (7%)	28 (6%)
Others	3 (2%)	9 (5%)	3 (2%)	15 (3%)
Settings currently practising*, n (%)				
Private practice	76 (51%)	70 (42%)	82 (52%)	228 (48%)
Public hospital	52 (35%)	64 (39%)	55 (35%)	171 (36%)
Private hospital	33 (22%)	40 (24%)	37 (23%)	110 (23%)
Sports teams	16 (11%)	16 (10%)	16 (10%)	48 (10%)
Aged care	10 (7%)	14 (8%)	7 (4%)	31 (7%)
Others	7 (5%)	8 (5%)	5 (3%)	20 (4%)
Involvement in other professional activities*, n (%)				
Teaching physiotherapy students	47 (31%)	63 (38%)	47 (30%)	157 (33%)
Research	36 (24%)	44 (27%)	33 (21%)	113 (24%)
Teaching continuing education courses	26 (17%)	37 (22%)	29 (18%)	92 (119%)
Others	2 (1%)	1 (1%)	3 (2%)	6 (1%)
None	75 (50%)	77 (47%)	82 (52%)	234 (49%)

* Total percentage exceeds 100% as participants could select multiple responses.

LBP, low back pain; n, number of participants in each group; N, total number of participants.

### Participants’ knowledge about guidelines and influence of evidence on practice

93% of physiotherapists reported that imaging guidelines somewhat (or greater) influence their practice and 89% of physiotherapists reported that evidence on electrotherapy somewhat (or greater) influences their practice (table 4).

**Table 4:** Knowledge about guidelines and influence of evidence on practice

Knowledge about evidence on low back pain (N=473)	Original recommendation (n=150)	Optimised recommendation (n=165)	No recommendation (n=158)	Total (N=473)
Knowledge about imaging guidelines for low back pain, n (%)				
Extremely knowledgeable	60 (40%)	53 (32%)	65 (41%)	178 (38%)
Very knowledgeable	64 (43%)	75 (46%)	58 (37%)	197 (42%)
Moderately knowledgeable	18 (12%)	30 (18%)	25 (16%)	73 (15%)
Slightly knowledgeable	8 (5%)	6 (4%)	10 (6%)	24 (5%)
Not knowledgeable at all	0 (0%)	1 (1%)	0 (0%)	1 (0.5%)
Knowledge about evidence on the effectiveness of electrotherapy for low back pain, n (%)				
Extremely knowledgeable	43 (29%)	49 (30%)	50 (32%)	142 (30%)
Very knowledgeable	74 (49%)	68 (41%)	73 (46%)	215 (45%)
Moderately knowledgeable	27 (18%)	40 (24%)	26 (17%)	93 (20%)
Slightly knowledgeable	4 (3%)	7 (4%)	7 (4%)	18 (4%)
Not knowledgeable at all	2 (1%)	1 (1%)	2 (1%)	5 (1%)
Imaging guidelines for low back pain influences practice, n (%)				
A great deal	57 (38%)	46 (28%)	52 (33%)	155 (33%)
A fair amount	63 (42%)	80 (49%)	58 (37%)	201 (42%)
Somewhat	23 (15%)	28 (17%)	33 (21%)	84 (18%)
Slightly	5 (3%)	8 (5%)	12 (8%)	25 (5%)
Not at all	2 (1%)	3 (2%)	3 (2%)	8 (2%)
Evidence on the effectiveness of electrotherapy for low back pain influences practice, n (%)				
A great deal	57 (38%)	58 (35%)	66 (42%)	181 (38%)
A fair amount	61 (41%)	62 (38%)	59 (37%)	182 (39%)
Somewhat	20 (13%)	25 (15%)	14 (9%)	59 (12%)
Slightly	5 (3%)	6 (4%)	8 (5%)	19 (4%)
Not at all	7 (5%)	14 (9%)	11 (7%)	32 (7%)

N, total number of participants; n, number of participants in each group.

### Physiotherapists’ intentions to refer for imaging or use electrotherapy for LBP (primary outcome)

Across all vignettes, no statistically significant differences were found in intentions to refer for imaging or use electrotherapy between those who received *Choosing Wisely recommendations* versus *no recommendation*, or between those who received *optimised* versus *original recommendations* (table 5). Intentions to use electrotherapy were low across all vignettes (range 12%–17%). However, intentions to use imaging varied, with some physiotherapists intending to use imaging when it was not indicated (vignettes 1 and 2) and some not intending to use imaging when it was indicated (vignette 3). We also did not find any statistically significant differences in the intentions to refer for imaging or use electrotherapy among physiotherapists when poor quality responses were excluded (online supplemental appendix 8). Online supplemental appendix 9 also shows the frequency and proportion of imaging and electrotherapy modalities chosen by participants in each of the three vignettes.

**Table 5:** Physiotherapists’ intentions to refer for imaging and use electrotherapy for low back pain

	Original	Optimised	No recommendation	Recommendation versus no recommendation (OR, 95% CI)	Optimised versus original (OR, 95% CI)
Vignette 1 (N=473)	n=150	n=165	n=158		
Imaging (inappropriate)	90 (60%)	82 (59%)	86 (54%)	0.9 (0.6 to 1.4)	0.7 (0.4 to 1.0)
Electrotherapy (inappropriate)	24 (16%)	21 (13%)	23 (15%)	1.0 (0.6 to 1.7)	0.8 (0.4 to 1.5)
Vignette 2 (N=467)	n=147	n=162	n=158		
Imaging (inappropriate)	81 (54%)	91 (55%)	86 (54%)	0.9 (0.6 to 1.4)	1.0 (0.7 to 1.7)
Electrotherapy (inappropriate)	21 (14%)	23 (14%)	23 (15%)	0.9 (0.5 to 1.7)	1.0 (0.5 to 1.9)
Vignette 3 (N=449)	n=146	n=147	n=156		
Imaging (appropriate)	90 (60%)	98 (59%)	106 (67%)	0.7 (0.5 to 1.0)	0.9 (0.6 to 1.6)
Electrotherapy (inappropriate)	29 (19%)	22 (13%)	23 (15%)	1.1 (0.7 to 2.0)	0.7 (0.4 to 1.2)

N, total number of participants in each vignette.

### Physiotherapists’ intentions to provide other treatments for LBP (secondary outcome)

Across all vignettes, there were no statistically significant differences in physiotherapists’ intention to provide other treatments for LBP when comparing those who received the *Choosing Wisely recommendations* versus *no recommendation* and those who received the *optimised* versus *original* Choosing Wisely recommendations (online supplemental appendix 10).

### Effect of Choosing Wisely recommendations on the decision-making process (secondary outcome)

Among participants randomised to one of the recommendation groups, 63% (n=179) reported feeling at least a ‘fair amount’ influenced by the recommendations (original recommendation—69%, n=95 and optimised recommendation—57%, n=84) (table 6).

**Table 6:** The influence of Choosing Wisely recommendations on decision-making in the vignettes

N=285	Original recommendation (n=138)	Optimised recommendation (n=147)	Total n (%)
A great deal	61 (44%)	49 (33%)	110 (39%)
A fair amount	34 (25%)	35 (24%)	69 (24%)
Somewhat	15 (11%)	22 (15%)	37 (13%)
Slightly	8 (6%)	13 (9%)	21 (7%)
Not at all	20 (14%)	28 (19%)	48 (17%)

n, number of participants in each group; N, total number of participants.

### Physiotherapists’ familiarity and agreement with the Choosing Wisely recommendations (secondary outcome)

Most physiotherapists were ‘moderately’ (or more) familiar with the Choosing Wisely recommendations for LBP (n=344, 79%). Most physiotherapists strongly agreed or somewhat agreed with the Choosing Wisely recommendations against imaging for low back (n=363, 83%) and against electrotherapy for LBP (n=348, 80%) (table 7).

**Table 7:** Familiarity and agreement with the APA Choosing Wisely recommendations for low back pain

Variables	n (%)
Familiarity with the APA Choosing Wisely recommendations for low back pain, N=435	
Extremely familiar	95 (22%)
Very familiar	147 (34%)
Moderately familiar	102 (23%)
Slightly familiar	32 (7%)
Not familiar at all	59 (14%)
Agreement with the Choosing Wisely recommendations against imaging for low back pain, N=435	
Strongly agree	192 (44%)
Somewhat agree	171 (39%)
Neither agree nor disagree	48 (11%)
Somewhat disagree	20 (5%)
Strongly disagree	4 (1%)
Agreement with the Choosing Wisely recommendations against electrotherapy for low back pain, N=435	
Strongly agree	177 (41%)
Somewhat agree	171 (39%)
Neither agree nor disagree	63 (14%)
Somewhat disagree	18 (4%)
Strongly disagree	6 (1%)

APA, Australian Physiotherapy Association; N, total number of participants; n, number of participants in each group.

## Discussion

Our study explored the effect of the Choosing Wisely recommendations on physiotherapists’ intentions to refer patients for imaging or use electrotherapy for LBP. We found no statistically significant differences in physiotherapists’ intentions to refer for imaging or use electrotherapy for LBP between those who received the Choosing Wisely recommendations (either in their original or optimised form) and those who did not. This finding is surprising given that most physiotherapists reported that imaging guidelines and evidence on electrotherapy for LBP substantially influence their clinical practice. The recommendations also did not affect physiotherapists’ intentions to use other treatments in the management of people with LBP.

A major strength of the study is its randomised design, which allowed for a more accurate assessment of the recommendations’ effects on physiotherapists’ intentions to refer for imaging and use electrotherapy for LBP. This design offers a clearer evaluation compared with our previous Best-Worst Scaling study involving 215 physiotherapists, which assessed the effect of language on physiotherapists’ willingness to follow the APA Choosing Wisely recommendations.^13^ Another strength is that we did not exclude participants based on their age, gender, clinical experience or area of specialisation. This enabled us to capture a diverse range of responses on how the Choosing Wisely recommendations impacted physiotherapists’ intentions to refer for imaging and use electrotherapy for LBP. Demographic characteristics of participants who exited the survey before responding to the first vignette were similar to those who completed the survey (online supplemental appendix 7); thus, our sample is representative of the physiotherapists who were initially willing to complete the survey. The dropout rates for participants who exited the survey after randomisation were low; hence, our study has a low risk of attrition bias.

The primary limitation of this study is that it was an online scenario-based experiment; it is possible that physiotherapists’ decision-making during face-to-face consultations could differ from the outcomes observed in this study. Another limitation is that the test and treatment options presented in the vignette were not randomised. This may have encouraged participants to select the first few options listed and potentially skewed the results towards those options.

An important consideration in interpreting these findings is the characteristics of the participants in our study. First, most participants had good experience managing patients with LBP, with 43% of physiotherapists seeing 6–50 patients with LBP in the past year and 52% seeing more than 50 in the past year. This experience may have meant physiotherapists had established routines in managing patients with LBP and were less likely to change their practice based on Choosing Wisely recommendations.^22^ In contrast, a sample of less experienced physiotherapists may have been more receptive to practice change after reading the recommendations.^12^ Second, most participants were from the USA (31%), UK (29%) and Australia (24%), countries with vastly different health systems. These differences likely influence how physiotherapists interpret and apply practice recommendations, could have possibly reduced the impact of the standardised Choosing Wisely recommendations.

The findings of our study suggest that while physiotherapists generally support the Choosing Wisely recommendations, merely presenting the recommendations to physiotherapists, even with modified language to be clearer and more positive, is not sufficient to change physiotherapists’ intentions about treatment decisions. Many physiotherapists in our study reported that the Choosing Wisely recommendations influenced their decision-making in the vignettes and indicated high familiarity and agreement with them. However, when they were presented with both original and modified versions of two APA Choosing Wisely recommendations targeting LBP, no significant change was observed in their intentions to refer for imaging or use electrotherapy. This contrasts with our previous qualitative study (n=19 physiotherapists) which explored attitudes, views and beliefs towards the APA original Choosing Wisely recommendations. This study identified that the language used in the recommendations was considered a barrier to their use.^12^ Similarly, our Best-Worst Scaling study involving 215 physiotherapists, assessed the effect of language on physiotherapists’ willingness to follow the APA Choosing Wisely recommendations.^13^ We modified the six original recommendations based on four language characteristics (level of detail, strength-qualified/unqualified, framing and alternatives to low-value care) to create 60 recommendations.^13^ The study found that modifications to the language of the recommendations, such as providing more detail, offering alternatives to low-value care and positively framing the recommendations, increased physiotherapists’ willingness to adopt them.^13^

Variations in findings across these studies may be due to differences in study methods. In the qualitative study, physiotherapists were asked to discuss barriers to using the recommendations, with the language of the recommendations identified as one of the barriers.^12^ In the Best-Worst Scaling study, physiotherapists rated recommendations based on their willingness to follow them, without any additional context.^13^ Meanwhile, in the current study, physiotherapists were presented with three hypothetical clinical scenarios that required more complex decision-making.

Our study revealed that while physiotherapists showed a high level of familiarity and agreement with the Choosing Wisely recommendations, the recommendations did not influence their intentions to refer for imaging or use electrotherapy for LBP. A possible explanation for the lack of effect in this current study is that awareness and agreement with guidelines are not sufficient to alter clinician intentions. Since intentions are behaviour-specific and can vary depending on the action and environment, it is important to understand how these factors interact.^23^ Clinicians’ decisions are shaped by elements such as cognitive bias,^24^ patient expectations,^25 26^ organisational culture,^27^ lack of time^28^ and clinical judgement,^22^ highlighting the complexity of behaviour change and the need for strategies that address both intentions and the context in which care is provided. Hence, to effectively drive change, it is essential to assess both current behaviours and the intentions behind them while considering key contextual factors that influence decision-making.

Modifying the language to make the Choosing Wisely recommendations clearer and more positive did not change physiotherapists’ clinical behaviour. Additional strategies beyond changing the language of the recommendations may be necessary to effectively alter their practice patterns regarding referrals for imaging or the use of electrotherapy for LBP. Trials that evaluate comprehensive, multicomponent implementation strategies for integrating the Choosing Wisely recommendations into clinical practice are needed. The implementation strategies could include a combination of two or more of the following components: educational meetings and training, workflow adjustments, decision support tools, reminders and ongoing feedback to translate evidence-based guidelines into practice to improve patient care. Studies show that such tailored, multi-component interventions positively impact awareness, knowledge and use of clinical practice guidelines.^29^,^32^

Future research could also explore how biomedical versus biopsychosocial orientations influence physiotherapists’ decision-making and response to Choosing Wisely recommendations. Understanding this could help improve guideline adherence, clinical outcomes and the implementation of evidence-based practices to optimise patient care.^33 34^ Another avenue for future research is further optimising the Choosing Wisely recommendations to increase their impact. For recommendations targeting LBP management, this could involve integrating information on red flags or clarifying imaging-related recommendations to improve decision-making and adherence to guidelines.

## Conclusion

These results suggest that simply presenting Choosing Wisely recommendations to physiotherapists does not influence their intentions to refer for imaging or use electrotherapy for LBP even if the language of the recommendations is optimised. There is a need for studies that evaluate multicomponent implementation strategies to implement these recommendations to align physiotherapists’ practices more closely with evidence-based guidelines.

## Supplementary material

10.1136/bmjopen-2024-097202 online supplemental file 1

## Data Availability

Data are available upon reasonable request.
