# Supplementary material for: Effectiveness of Choosing Wisely recommendations in reducing physiotherapists’ intentions to refer for imaging and use electrotherapy for low back pain: a randomised controlled experiment
Source: BMJ Open. 2025 Jun 26;15(6):e097202. doi: 10.1136/bmjopen-2024-097202 (PMC12207150; doi:10.1136/bmjopen-2024-097202)
Supplement: online supplemental file 1 [file bmjopen-15-6-s001.docx]

**Appendix 1: CONSORT CHECKLIST**


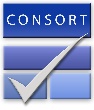
CONSORT 2010 checklist of information to include when reporting a randomised trial*

| Section/Topic | Item No | Checklist item | Reported on page No |
| --- | --- | --- | --- |
| Title and abstract | | | |
|  | 1a | Identification as a randomised trial in the title | 1 and 3 |
|  | 1b | Structured summary of trial design, methods, results, and conclusions (for specific guidance see CONSORT for abstracts) | 3 |
| Introduction | | | |
| Background and objectives | 2a | Scientific background and explanation of rationale | 5 |
|  | 2b | Specific objectives or hypotheses | 6 |
| Method | | | |
| Trial design | 3a | Description of trial design (such as parallel, factorial) including allocation ratio | 6-7 |
|  | 3b | Important changes to methods after trial commencement (such as eligibility criteria), with reasons | n/a |
| Participants | 4a | Eligibility criteria for participants | 6-7 |
|  | 4b | Settings and locations where the data were collected | 6-7 |
| Interventions | 5 | The interventions for each group with sufficient details to allow replication, including how and when they were actually administered | 7-9, Appendix 5 |
| Outcomes | 6a | Completely defined pre-specified primary and secondary outcome measures, including how and when they were assessed | 9-11 |
|  | 6b | Any changes to trial outcomes after the trial commenced, with reasons | n/a |
| Sample size | 7a | How sample size was determined | 11 |
|  | 7b | When applicable, explanation of any interim analyses and stopping guidelines | n/a |
| Randomisation: |  |  |  |
| Sequence generation | 8a | Method used to generate the random allocation sequence | 6-7 |
|  | 8b | Type of randomisation; details of any restriction (such as blocking and block size) | 6-7 |
| Allocation concealment mechanism | 9 | Mechanism used to implement the random allocation sequence (such as sequentially numbered containers), describing any steps taken to conceal the sequence until interventions were assigned | n/a |
| Implementation | 10 | Who generated the random allocation sequence, who enrolled participants, and who assigned participants to interventions | 6-7 |
| Blinding | 11a | If done, who was blinded after assignment to interventions (for example, participants, care providers, those assessing outcomes) and how | n/a |
|  | 11b | If relevant, description of the similarity of interventions | 7-8  Appendix 5 |
| Statistical methods | 12a | Statistical methods used to compare groups for primary and secondary outcomes | 12 |
|  | 12b | Methods for additional analyses, such as subgroup analyses and adjusted analyses | 12 |
| Results | | | |
| Participant flow (a diagram is strongly recommended) | 13a | For each group, the numbers of participants who were randomly assigned, received intended treatment, and were analysed for the primary outcome | 13, Figure 1 |
|  | 13b | For each group, losses and exclusions after randomisation, together with reasons | 13, Figure 1 |
| Recruitment | 14a | Dates defining the periods of recruitment and follow-up | 7 |
|  | 14b | Why the trial ended or was stopped | n/a |
| Baseline data | 15 | A table showing baseline demographic and clinical characteristics for each group | Table 2 |
| Numbers analysed | 16 | For each group, number of participants (denominator) included in each analysis and whether the analysis was by original assigned groups | Table 3-5 |
| Outcomes and estimation | 17a | For each primary and secondary outcome, results for each group, and the estimated effect size and its precision (such as 95% confidence interval) | Table 3-5 |
|  | 17b | For binary outcomes, presentation of both absolute and relative effect sizes is recommended | n/a |
| Ancillary analyses | 18 | Results of any other analyses performed, including subgroup analyses and adjusted analyses, distinguishing pre-specified from exploratory | n/a |
| Harms | 19 | All important harms or unintended effects in each group (for specific guidance see CONSORT for harms) | n/a |
| Discussion | | | |
| Limitations | 20 | Trial limitations, addressing sources of potential bias, imprecision, and, if relevant, multiplicity of analyses | 22-23 |
| Generalisability | 21 | Generalisability (external validity, applicability) of the trial findings | 22-23 |
| Interpretation | 22 | Interpretation consistent with results, balancing benefits and harms, and considering other relevant evidence | 22-24 |
| Other information | | |  |
| Registration | 23 | Registration number and name of trial registry | n/a |
| Protocol | 24 | Where the full trial protocol can be accessed, if available | n/a |
| Funding | 25 | Sources of funding and other support (such as supply of drugs), role of funders | 26 |

Citation: Schulz KF, Altman DG, Moher D, for the CONSORT Group. CONSORT 2010 Statement: updated guidelines for reporting parallel group randomised trials. BMC Medicine. 2010;8:18.
© 2010 Schulz et al. This is an Open Access article distributed under the terms of the Creative Commons Attribution License (<http://creativecommons.org/licenses/by/2.0>), which permits unrestricted use, distribution, and reproduction in any medium, provided the original work is properly cited.

*We strongly recommend reading this statement in conjunction with the CONSORT 2010 Explanation and Elaboration for important clarifications on all the items. If relevant, we also recommend reading CONSORT extensions for cluster randomised trials, non-inferiority and equivalence trials, non-pharmacological treatments, herbal interventions, and pragmatic trials. Additional extensions are forthcoming: for those and for up-to-date references relevant to this checklist, see [www.consort-statement.org](http://www.consort-statement.org).

**Appendix 2: Welcome to the study**

**Welcome to the study investigating health professionals’ test and treatment choices for musculoskeletal pain!**

Thank you for your interest.

***What is the aim of this project?***
Researchers at the University of Sydney are doing this study to assess what tests and treatments health professionals provide to people with musculoskeletal pain presented in the clinical vignettes. 

***What does participation involve?***
Participation involves completing one brief survey that will take approximately 10-15 minutes. Our researchers take your privacy very seriously and all responses will be anonymous. You can also exit from the survey at any time.

A Participant Information Sheet is available [here](https://sydney.au1.qualtrics.com/CP/File.php?F=F_d0GL9StzWgYhAeq). You should review and retain this information sheet before proceeding. Please read it carefully before making up your mind about taking part. If you have any questions, please get in touch with a member of the research team using the phone numbers or emails listed in the information sheet. The University of Sydney Human Research Ethics Committee has approved this study (Protocol number: 2023/672).

Once you have read the Participant Information Sheet, please click the next button to get the consent form and start the survey.

***Who can participate?***

You must

- be over the age of 18

- be a registered health professional

Thank you for supporting this important research.

**Appendix 3: Participant Information Statement**

| 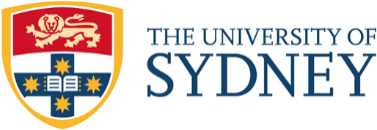 | |  | **School of Public Health Faculty of Medicine and Health** | |
| --- | --- | --- | --- | --- |
|  | ABN 15 211 513 464 | | |  |
|  | **Priti Kharel**  *Chief Investigator  PhD Candidate* | | | Room 10/048  Level 10 North, King George V Building Royal Prince Alfred Hospital  The University of Sydney  NSW 2050 AUSTRALIA  Telephone: +61 2 8627 6691  Facsimile: +61 2 8627 6262  Email: [priti.kharel@sydney.edu.au](mailto:priti.kharel@sydney.edu.au)  Web: <http://www.sydney.edu.au> |

**Health professionals’ test and treatment choices for musculoskeletal pain!**

PARTICIPANT INFORMATION STATEMENT

1. **What is this study about?**

You are invited to take part in a research study about health professionals’ test and treatment choices for musculoskeletal pain. This Participant Information Statement provides essential details about the research study. Knowing what is involved will help you decide if you want to take part. Please read this sheet carefully and ask questions about anything that is unclear or that you want to know more about.

Participation in this research study is voluntary.

By giving your consent to take part in this study you are telling us that you:

- Understand what you have read.
- Agree to take part in the research study as outlined below.
- Agree to the use of your personal information as described.

You will be given the opportunity to download this Participant Information Statement to keep.

1. **Who is running the study?**

The study is being carried out by the following researchers at the University of Sydney:

- Priti Kharel, PhD Candidate
- Dr Joshua Zadro, Research Fellow
- Prof Christopher Maher, Director of Institute for Musculoskeletal Health
- Dr Giovanni Ferreira, Research Fellow
- Mr Andrew Gamble, Physiotherapist and PhD Candidate

1. **What will the study involve for me?**

If you agree to participate in the study, you will be directed to first answer demographic questions, such as your age, gender, qualification(s), years since graduation, clinical area of interest and average number of musculoskeletal pain patient encounters per week, etc.

After this, we will show you some hypothetical vignettes of patients with musculoskeletal pain who have come to seek diagnosis and treatment from a health professional. To get accurate responses to the hypothetical vignette it is important that you imagine yourself being the health professional seeing the patient. We want you to think carefully about what tests or treatments you would provide the patient and answer some questions about it. At the end of the study, if you desire any more information, relevant contact details will be provided.

If the study causes you distress at any time or makes you feel uncomfortable, you may withdraw participation. More details are provided below about withdrawal and risks associated with the study.

1. **How much of my time will the study take?**

The study is expected to take around 15 minutes to complete.

1. **Do I have to be in the study? Can I withdraw from the study once I've started?**

Being in this study is completely voluntary and you do not have to take part. Your decision whether to participate will not affect your current or future relationship with the researchers or anyone else at the University of Sydney.

If you decide to take part in the study and then change your mind later, you are free to withdraw at any time. You can do this by not submitting the questionnaire and just exiting part way through. If you do, any previous responses made by you will be destroyed and not used in any analysis.

Submitting your completed questionnaire is an indication of your consent to participate in the study. Once you have submitted it, your responses cannot be withdrawn because they are anonymous and therefore, we will not be able to tell which one is yours.

1. **Are there any risks or costs associated with being in the study?**

There are no potential risks to participating in this study. The survey does not address topics of a sensitive nature. The only anticipated consequence of participation is time, with the online survey taking approximately 15 minutes to complete.

1. **Are there any benefits associated with being in the study?**

We cannot and do not guarantee that you will receive any direct benefits from being in the study. However, you may find the information helpful to think about while treating patients with musculoskeletal pain.

1. **What will happen to information about me that is collected during the study?**

By providing your consent, you are agreeing to us collecting personal information about you for the purpose of this research study. Your information will only be used for the purposes outlined in this Participant Information Statement unless you consent otherwise.

Your survey responses will be stored securely, and your identity/information will be kept strictly confidential, except as required by law. Study findings may be published, but you will not be individually identifiable in these publications*.*

The data collected in this project will be securely kept in perpetuity and may be used in future research studies, however, all identifiable data will be removed. Ethical approval will be obtained before using the data in future projects.

1. **What if I would like further information about the study?**

Please feel free to contact Priti Kharel to discuss the research further or if you have any additional questions

- Priti Kharel, PhD candidate, email: [priti.kharel@sydney.edu.au](mailto:priti.kharel@sydney.edu.au)

1. **Will I be told the results of the study?**

You have a right to receive feedback about the overall results of this study. You can tell us that you wish to receive feedback by emailing or calling Priti Kharel using the above details. You will also be reminded on this at the end of the survey. This feedback will be a one-page summary regarding the overall findings of the study. You will receive it after the study is finished. Feedback regarding personal results will not be available, as all data collected is stored as anonymous and cannot be traced back to you.

1. **What if I have a complaint or any concerns about the study?**

Research involving humans in Australia is reviewed by an independent group of people called the Human Research Ethics Committee (HREC). The ethical aspects of this study have been approved by the HREC of the University of Sydney *[2023/672].* As part of this process, we have agreed to carry out the study according to the *National Statement on Ethical Conduct in Human Research (2007).* This statement has been developed to protect people who agree to take part in research studies.

If you are concerned about the way this study is being conducted or you wish to make a complaint to someone independent from the study, please contact the university using the details outlined below. Please quote the study title and protocol number.

The Manager, Ethics Administration, University of Sydney:

- - **Telephone:** +61 2 8627 8176
  - **Email:** [human.ethics@sydney.edu.au](mailto:human.ethics@sydney.edu.au)
  - **Fax:** +61 2 8627 8177

This information sheet is for you to keep.

| 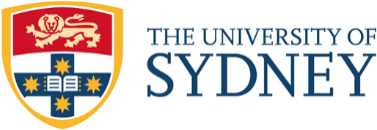**Appendix 4: Participant consent form** | | | |
| --- | --- | --- | --- |
|  | |  | **School of Public Health Faculty of Medicine and Health** |
|  | ABN 15 211 513 464 | |  |
|  | **Priti Kharel**  *Chief Investigator  PhD Candidate* | | Room 10/048  Level 10 North, King George V Building Royal Prince Alfred Hospital  The University of Sydney  NSW 2050 AUSTRALIA  Telephone: +61 2 8627 6691  Facsimile: +61 2 8627 6262  Email: [priti.kharel@sydney.edu.au](mailto:priti.kharel@sydney.edu.au)  Web: <http://www.sydney.edu.au> |

**Health professionals’ test and treatment choices for musculoskeletal pain**

1. In giving my consent, I acknowledge that:

| ✓ | I have read the Participant Information Statement and have been given the opportunity to discuss the study and my involvement in it with the researcher/s. |
| --- | --- |
| ✓ | The procedures required and time involved (including any inconvenience, risk, discomfort or side effect, and their implications) have been explained to me, and my questions about the project have been answered to my satisfaction. |
| ✓ | I understand that participation is voluntary. I am under no obligation to consent. |
| ✓ | I understand that I can withdraw from the study at any time, without providing a reason and without suffering any penalty. This will not affect my relationship with the researcher/s or university. |
| ✓ | I understand that my involvement is strictly confidential and no information about me will be used in any way that reveals my identity. |
| ✓ | I understand that data from this study may be used again for future research purposes, but that all data is strictly confidential and no information about me will be used in any way that reveals my identity. |

- Yes, I would be happy to go on and complete the survey
- No, I would prefer not to complete the survey

**Appendix 5: Survey questions**

**Section 1: Screening questions**

Screening questions for participants from **social media**

| 1. Are you a registered physiotherapist?  - Yes - No *(excluded)*  1. How many patients with low back pain have you treated in the last 12 months?  - 0 *(excluded)* - 1-5 - 6-50 - 51-100 - >100 |
| --- |

Adjusted screening questions for participants from **market research company**:

| 1. Are you a registered health professional?  - Yes - No *(excluded)*  1. Which type of health professional are you registered as? If multiple, please select the one you currently spend the most time practising as  - Doctor *(excluded)* - Medical Specialist (e.g. surgeon, rheumatologist) *(excluded)* - Nurse *(excluded)* - Physiotherapist - Psychologist *(excluded)* - Other (please specify) *(excluded)*  1. Do you manage patients with any of the following conditions? Please select all that apply.  - Neck pain *(excluded)* - Shoulder or upper limb pain *(excluded)* - Hip pain *(excluded)* - Back pain - Knee or lower limb pain *(excluded)* - None of the above *(excluded)*  1. How many patients with low back pain have you treated in the last 12 months?  - 0 *(excluded)* - 1-5 - 6-50 - 51-100 - >100 |
| --- |

**Section 2: Survey**

**Now some quick questions about you...**

1. Please indicate your gender:

- Male
- Female
- Non-binary/third gender
- Prefer not to say

1. Please indicate your age:

- <20
- 20-29
- 30-39
- 40-49
- 50+

1. What qualifications do you have relevant to physiotherapy? (select as many as apply)

- Bachelors
- Masters
- PhD
- Titling
- Specialisation
- Other (please specify) ____________________________

1. In which country(ies) did you receive your qualification(s)? (select as many as apply)

- Australia
- USA
- UK
- Canada
- Ireland
- Brazil
- Other (please specify) __________________

1. In which country do you practice:

- Australia
- USA
- UK
- Canada
- Ireland
- Brazil
- Other (please specify) __________________

1. What do you consider your clinical area(s) of interest? (select as many as apply)

- Cardiothoracic
- Continence and women's health
- Ergonomics and occupational health
- Gerontology
- Musculoskeletal
- Neurology
- Oncology
- Orthopaedics
- Paediatrics
- Sports
- Other (please specify) ____________________________

1. Which clinical setting(s) have you spent the most time practising in? (select as many as apply)

- Private practice
- Public hospital
- Private hospital
- Aged care
- Sports teams
- Other (please specify) ____________________________

1. Which clinical setting(s) are you **currently** practising in? (select as many as apply)

- Private practice
- Public hospital
- Private hospital
- Aged care
- Sports teams
- Other (please specify) ____________________________

1. Are you currently involved in any of the following professional activities? (select as many as apply)

- Research
- Teaching physiotherapy students
- Teaching continuing education courses
- Other (please specify) ____________________________
- None

1. How many years have you been practising as a physiotherapist?

- <1 year
- 1 - 5 years
- 6 - 10 years
- > 10 years

**Some clinical questions…**

1. How would you rate your knowledge in the following areas?

|  | Extremely knowledgeable | Very knowledgeable | Moderately knowledgeable | Slightly knowledgeable | Not knowledgeable at all |
| --- | --- | --- | --- | --- | --- |
| Imaging guidelines for low back pain |  |  |  |  |  |
| Evidence on the effectiveness of electrotherapy for low back pain |  |  |  |  |  |

1. How much do the following influence your practice?

|  | A great deal | A fair amount | Somewhat | Slightly | Not at all |
| --- | --- | --- | --- | --- | --- |
| Imaging guidelines for low back pain |  |  |  |  |  |
| Evidence on the effectiveness of electrotherapy for low back pain |  |  |  |  |  |

**Section 3: Randomisation (participants will then be randomised into one of the three groups below)**

**Group 1 receive the original Choosing Wisely recommendations for imaging and electrotherapy for low back pain**

Choosing Wisely is a global campaign, launched in 2012, that aims to encourage conversations between patients and healthcare providers about low-value care. Low-value care is care that provides little-to-no-benefit, causes harm, or diverts resources from care that is evidence-based, safe and truly necessary (high-value care).

Since its launch, the campaign has been endorsed by over 250 professional societies around the world, including the Australian Physiotherapy Association (APA), with each society publishing their own list of recommendations against the use of low-value tests and treatments. In 2015, the APA published 6 recommendations against the use of test and treatments that are potentially low-value and frequently provided by physiotherapists.

Two of the APA recommendations are relevant to the management of low back pain:

1. **Don't request imaging for patients with non-specific low back pain and no indicators of a serious cause for low back pain.**
2. **Avoid using electrotherapy modalities in the management of patients with low back pain.**

You will now be shown three hypothetical vignettes of patients with low back pain visiting a physiotherapist. Consider the two Choosing Wisely recommendations when responding to the vignettes. **We want you to imagine you are the physiotherapist responsible for treating these patients and indicate what test and treatment options you would consider at any point in the patient's episode of care.**

You are free to select as many or as few options as you want.

**Section 4: Clinical vignettes**

**Hypothetical vignette 1**

**History:** A 50-year-old man has been suffering from low back pain for the past 6 weeks. He comes to see you with direct access. The pain started after he helped his 25-year-old son renovate a house. He did not lift any heavy objects. The pain is continuous and radiates to the left buttock. He called in sick to work due to the pain and has still not gone back to work. He is an electrician in a hardware store. The pain has not reduced over the past 6 weeks despite the fact that he lies down regularly. He loves to play golf, but he has not tried to play golf since he developed low back pain, he believes that playing golf will exacerbate the problem. He takes paracetamol for the pain as necessary, varying from 0-5 tablets per day.

**Physical Exam:** Vital signs: blood pressure 110/70 mmHg and pulse rate 60. During range of motion testing, he experiences some pain during back extension and lateral flexion, particularly to the right (these are not noticeably limited), but flexion is nearly impossible. The straight leg raise (SLR) test on the left provokes low back pain at 80 degrees. He is not willing to lift a 20-lb weight from the floor, because he expects it will further damage his back. He assesses his control over the pain as low and lacks confidence that he could control the pain. Palpation and accessory motion testing did not reproduce low back pain symptoms; however, tenderness was noted diffusely and bilaterally from L1 to L5.

Please choose from the following list of tests and treatments that you believe would be appropriate for the patient described in vignette 1

- Refer for Bone Scan
- Refer for CT
- Refer for magnetic resonance imaging
- Refer for radiographs
- Refer for ultrasound
- Refer to the medical doctor and maintain physiotherapy treatment
- Refer to the medical doctor without intervention
- Advice to maintain an upright posture during bending and lifting
- Advice to pursue or maintain an active lifestyle
- Aerobic and fitness exercises
- Back school (a booklet that includes instructions on the home exercise program)
- Bed rest
- Directional preference exercises: extension
- Directional preference exercises: flexion
- Gradual exercise exposure
- Ice or heat
- Interferential current or transcutaneous electrical nerve stimulation (TENS)
- Laser or ultrasound
- Lumbar brace or corset
- Lumbar stabilisation exercises
- Mechanical traction
- Neurodynamic mobilisation
- Pain neuroscience education (e.g. "pain does not equal damage")
- Psychologically informed physiotherapy (behavioural therapy + regular physiotherapy)
- Spinal manipulation (thrust)
- Spinal mobilisation (non-thrust)
- Other (please specify) __________________________________________________

**Hypothetical vignette 2**

**History:** A 28-year-old woman has suffered from low back pain on and off for 45 days. Low back pain started insidiously and does not appear to change with physical activity or posture. She works as a schoolteacher. She sits all day. Her back pain is worse in the end of the day and at night. The pain does not radiate to the legs; it is located on the low back and left buttock area. She has been married for five years; she has no children and is trying to become pregnant. Her medical history is unremarkable, except that her period is 6 weeks late and she reports a pinkish vaginal discharge. She is taking 200 mg ibuprofen four times a day for the back pain.

**Physical exam**: Vital signs: blood pressure 90/60 mm Hg and pulse rate 102. Back range of motion is full of pain at end range of flexion. Straight leg raise stretch the hamstrings bilaterally at 80 degrees of hip flexion. The neurological exam is within normal limits. Palpation and accessory motion testing did not reproduce low back pain symptoms; however, tenderness was noted from the L3 to the L5 lumbar vertebrae with PA pressure.

Please choose from the following list of tests and treatments that you believe would be appropriate for the patient described in vignette 2

- Refer for Bone Scan
- Refer for CT
- Refer for magnetic resonance imaging
- Refer for radiographs
- Refer for ultrasound
- Refer to the medical doctor and maintain physical therapy treatment
- Refer to the medical doctor without intervention
- Advice to maintain an upright posture during bending and lifting
- Advice to pursue or maintain an active lifestyle
- Aerobic and fitness exercises
- Back school (a booklet that includes instructions on the home exercise program)
- Bed rest
- Directional preference exercises: extension
- Directional preference exercises: flexion
- Gradual exercise exposure
- Ice or heat
- Interferential current or transcutaneous electrical nerve stimulation (TENS)
- Laser or ultrasound
- Lumbar brace or corset
- Lumbar stabilisation exercises
- Mechanical traction
- Neurodynamic mobilisation
- Pain neuroscience education (e.g. "pain does not equal damage")
- Psychologically informed physiotherapy (behavioural therapy + regular physiotherapy)
- Spinal manipulation (thrust)
- Spinal mobilisation (non-thrust)
- Other (specify)___________________________________________________

**Hypothetical vignette 3**

**History:** A 55-year-old male is referred to physical therapy complaining of low back pain. He is a heavy smoker. The pain started suddenly 2 weeks ago. The patient has noticed significant weight loss; he mentioned that he dropped two sizes. Pain is constant and does not seem to be related to changes in positions or physical activities. Pain is localised at the L1- L2 level and not referring to the lower extremities. The patient reported that the pain severity peaks at midnight with excessive, unusual sweating.

**Physical examination:** Heart rate was 104 beats per minute, blood pressure 145/60 mm Hg and he had an elevated body temperature. Lumbar range of motion was within normal limits with discomfort at the end range. Accessory motion testing did not exacerbate the patient’s symptoms. Tenderness was noted at the L1-2 level.

Please choose from the following list of tests and treatments that you believe would be appropriate for the patient described in vignette 3

- Refer for Bone Scan
- Refer for CT
- Refer for magnetic resonance imaging
- Refer for radiographs
- Refer for ultrasound
- Refer to the medical doctor and maintain physical therapy treatment
- Refer to the medical doctor without intervention
- Advice to maintain an upright posture during bending and lifting
- Advice to pursue or maintain an active lifestyle
- Aerobic and fitness exercises
- Back school (a booklet that includes instructions on the home exercise program)
- Bed rest
- Directional preference exercises: extension
- Directional preference exercises: flexion
- Gradual exercise exposure
- Ice or heat
- Interferential current or transcutaneous electrical nerve stimulation (TENS)
- Laser or ultrasound
- Lumbar brace or corset
- Lumbar stabilisation exercises
- Mechanical traction
- Neurodynamic mobilisation
- Pain neuroscience education (e.g. "pain does not equal damage")
- Psychologically informed physiotherapy (behavioural therapy + regular physiotherapy)
- Spinal manipulation (thrust)
- Spinal mobilisation (non-thrust)
- Other (specify)__________________________________________________

**Section 5: Information about Choosing Wisely recommendations**

1. Please rate your agreement with the following Choosing Wisely recommendations for physiotherapists.

|  | Strongly agree | Somewhat  agree | Neither agree nor disagree | Somewhat  disagree | Strongly disagree |
| --- | --- | --- | --- | --- | --- |
| **Don’t request imaging for patients with non-specific low back pain and no indicators of a serious cause for low back pain** |  |  |  |  |  |
| **Avoid using electrotherapy modalities in the management of patients with low back pain** |  |  |  |  |  |

1. How familiar were you with the Australia Physiotherapy Association’s Choosing Wisely recommendations prior to being invited to complete this survey?

- Extremely familiar
- Very familiar
- Moderately familiar
- Slightly familiar
- Not familiar at all

**Questions only for the groups that are allocated to receive the recommendations**

1. To what extent did the Choosing Wisely recommendations you were shown before the vignettes influence your decision making? Please rate on a Likert scale from 0 (completely unaffected) to 5 (highly affected).

- A great deal
- A fair amount
- Somewhat
- Slightly
- Not at all

1. If the recommendations affected or influenced your decision making, please provide a brief explanation of how the recommendations influenced your management choices. If no, kindly explain why the recommendations did not have an impact on your decision-making. (Please provide a free text response.)

___________________

1. In your own words, please describe what you liked about the recommendations you received? (Please provide a free text response.)

___________________

1. In your own words, please describe what you disliked about the recommendations you received? (Please provide a free text response.)

___________________

**Section 6: Results of this study**

You have a right to receive feedback about the overall results of this study. This feedback will be a one-page summary regarding the overall findings of the study.

Please feel free to contact Priti Kharel if you would like to receive overall feedback on the results of this study.

Priti Kharel, PhD Candidate, email: priti.kharel@sydney.edu.au phone: 02 8627 6782

**Group 2 receive the optimised Choosing Wisely recommendations for imaging and electrotherapy for low back pain**

Choosing Wisely is a global campaign, launched in 2012, that aims to encourage conversations between patients and healthcare providers about low-value care. Low-value care is care that provides little-to-no-benefit, causes harm, or diverts resources from care that is evidence-based, safe and truly necessary (high-value care).

Since its launch, the campaign has been endorsed by over 250 professional societies around the world, including the Australian Physiotherapy Association (APA), with each society publishing their own list of recommendations against the use of low-value tests and treatments. In 2015, the APA published 6 recommendations against the use of test and treatments that are potentially low-value and frequently provided by physiotherapists.

We then conducted a series of studies to optimise the wording of these recommendations, which are as follows:

1. **Physiotherapists should not request imaging for patients with non-specific low back pain and no indicators of a serious cause for low back pain as the findings are unlikely to positively guide management. Physiotherapists should instead explain why imaging is not required.**
2. **Physiotherapists should not use electrotherapy modalities in the management of patients with low back pain as they are not superior to placebo. Physiotherapists should instead give advice to stay active and reassurance.**

You will now be shown three hypothetical vignettes of patients with low back pain visiting a physiotherapist. Consider the two Choosing Wisely recommendations when responding to the vignettes. **We want you to imagine you are the physiotherapist responsible for treating these patients and indicate what test and treatment options you would consider at any point in the patient's episode of care.** You are free to select as many or few options as you want.

**Section 4: Clinical vignettes**

**Hypothetical vignette 1**

**History:** A 50-year-old man has been suffering from low back pain for the past 6 weeks. He comes to see you with direct access. The pain started after he helped his 25-year-old son renovate a house. He did not lift any heavy objects. The pain is continuous and radiates to the left buttock. He called in sick to work due to the pain and has still not gone back to work. He is an electrician in a hardware store. The pain has not reduced over the past 6 weeks despite the fact that he lies down regularly. He loves to play golf, but he has not tried to play golf since he developed low back pain, he believes that playing golf will exacerbate the problem. He takes paracetamol for the pain as necessary, varying from 0-5 tablets per day.

**Physical Exam:** Vital signs: blood pressure 110/70 mmHg and pulse rate 60. During range of motion testing, he experiences some pain during back extension and lateral flexion, particularly to the right (these are not noticeably limited), but flexion is nearly impossible. The straight leg raise test on the left provokes low back pain at 80 degrees. He is not willing to lift a 20-lb weight from the floor, because he expects it will further damage his back. He assesses his control over the pain as low and lacks confidence that he could control the pain. Palpation and accessory motion testing did not reproduce low back pain symptoms; however, tenderness was noted diffusely and bilaterally from L1 to L5.

Please choose from the following list of tests and treatments that you believe would be appropriate for the patient described in vignette 1

- Refer for Bone Scan
- Refer for CT
- Refer for magnetic resonance imaging
- Refer for radiographs
- Refer for ultrasound
- Refer to the medical doctor and maintain physiotherapy treatment
- Refer to the medical doctor without intervention
- Advice to maintain an upright posture during bending and lifting
- Advice to pursue or maintain an active lifestyle
- Aerobic and fitness exercises
- Back school (a booklet that includes instructions on the home exercise program)
- Bed rest
- Directional preference exercises: extension
- Directional preference exercises: flexion
- Gradual exercise exposure
- Ice or heat
- Interferential current or transcutaneous electrical nerve stimulation (TENS)
- Laser or ultrasound
- Lumbar brace or corset
- Lumbar stabilisation exercises
- Mechanical traction
- Neurodynamic mobilisation
- Pain neuroscience education (e.g. "pain does not equal damage")
- Psychologically informed physiotherapy (behavioural therapy + regular physiotherapy)
- Spinal manipulation (thrust)
- Spinal mobilisation (non-thrust)
- Other (specify) __________________________________________________

**Hypothetical vignette 2**

**History:** A 28-year-old woman has suffered from low back pain on and off for 45 days. Low back pain started insidiously and does not appear to change with physical activity or posture. She works as a schoolteacher. She sits all day. Her back pain is worse in the end of the day and at night. The pain does not radiate to the legs; it is located on the low back and left buttock area. She has been married for five years; she has no children and is trying to become pregnant. Her medical history is unremarkable, except that her period is 6 weeks late and she reports a pinkish vaginal discharge. She is taking 200 mg ibuprofen four times a day for the back pain.

**Physical exam**: Vital signs: blood pressure 90/60 mm Hg and pulse rate 102. Back range of motion is full of pain at end range of flexion. Straight leg raise stretch the hamstrings bilaterally at 80 degrees of hip flexion. The neurological exam is within normal limits. Palpation and accessory motion testing did not reproduce low back pain symptoms; however, tenderness was noted from the L3 to the L5 lumbar vertebrae with PA pressure.

Please choose from the following list of tests and treatments that you believe would be appropriate for the patient described in vignette 2

- Refer for Bone Scan
- Refer for CT
- Refer for magnetic resonance imaging
- Refer for radiographs
- Refer for ultrasound
- Refer to the medical doctor and maintain physical therapy treatment
- Refer to the medical doctor without intervention
- Advice to maintain an upright posture during bending and lifting
- Advice to pursue or maintain an active lifestyle
- Aerobic and fitness exercises
- Back school (a booklet that includes instructions on the home exercise program)
- Bed rest
- Directional preference exercises: extension
- Directional preference exercises: flexion
- Gradual exercise exposure
- Ice or heat
- Interferential current or transcutaneous electrical nerve stimulation (TENS)
- Laser or ultrasound
- Lumbar brace or corset
- Lumbar stabilisation exercises
- Mechanical traction
- Neurodynamic mobilisation
- Pain neuroscience education (e.g. "pain does not equal damage")
- Psychologically informed physiotherapy (behavioural therapy + regular physiotherapy)
- Spinal manipulation (thrust)
- Spinal mobilisation (non-thrust)
- Other (specify)___________________________________________________

**Hypothetical vignette 3**

**History:** A 55-year-old male is referred to physical therapy complaining of low back pain. He is a heavy smoker. The pain started suddenly 2 weeks ago. The patient has noticed significant weight loss; he mentioned that he dropped two sizes. Pain is constant and does not seem to be related to changes in positions or physical activities. Pain is localised at the L1- L2 level and not referring to the lower extremities. The patient reported that the pain severity peaks at midnight with excessive, unusual sweating.

**Physical examination:** Heart rate was 104 beats per minute, blood pressure 145/60 mm Hg and he had an elevated body temperature. Lumbar range of motion was within normal limits with discomfort at the end range. Accessory motion testing did not exacerbate the patient’s symptoms. Tenderness was noted at the L1-2 level.

Please choose from the following list of tests and treatments that you believe would be appropriate for the patient described in vignette 3

- Refer for Bone Scan
- Refer for CT
- Refer for magnetic resonance imaging
- Refer for radiographs
- Refer for ultrasound
- Refer to the medical doctor and maintain physical therapy treatment
- Refer to the medical doctor without intervention
- Advice to maintain an upright posture during bending and lifting
- Advice to pursue or maintain an active lifestyle
- Aerobic and fitness exercises
- Back school (a booklet that includes instructions on the home exercise program)
- Bed rest
- Directional preference exercises: extension
- Directional preference exercises: flexion
- Gradual exercise exposure
- Ice or heat
- Interferential current or transcutaneous electrical nerve stimulation (TENS)
- Laser or ultrasound
- Lumbar brace or corset
- Lumbar stabilisation exercises
- Mechanical traction
- Neurodynamic mobilisation
- Pain neuroscience education (e.g. "pain does not equal damage")
- Psychologically informed physiotherapy (behavioural therapy + regular physiotherapy)
- Spinal manipulation (thrust)
- Spinal mobilisation (non-thrust)
- Other (specify)__________________________________________________

**Section 5: Information about Choosing Wisely recommendations**

1. Please rate your agreement with the following Choosing Wisely recommendations for physiotherapists.

|  | Strongly agree | Somewhat  agree | Neither agree nor disagree | Somewhat  disagree | Strongly disagree |
| --- | --- | --- | --- | --- | --- |
| **Don’t request imaging for patients with non-specific low back pain and no indicators of a serious cause for low back pain** |  |  |  |  |  |
| **Avoid using electrotherapy modalities in the management of patients with low back pain** |  |  |  |  |  |

1. How familiar were you with the Australia Physiotherapy Association’s Choosing Wisely recommendations prior to being invited to complete this survey?

- Extremely familiar
- Very familiar
- Moderately familiar
- Slightly familiar
- Not familiar at all

**Questions only for the groups that are allocated to receive the recommendations**

1. To what extent did the Choosing Wisely recommendations you were shown before the vignettes influence your decision making? Please rate on a Likert scale from 0 (completely unaffected) to 5 (highly affected).

- A great deal
- A fair amount
- Somewhat
- Slightly
- Not at all

1. If the recommendations affected or influenced your decision making, please provide a brief explanation of how the recommendations influenced your management choices. If no, kindly explain why the recommendations did not have an impact on your decision-making. (Please provide a free text response)

___________________

1. In your own words, please describe what you liked about the recommendations you received? (Please provide a free text response.)

___________________

1. In your own words, please describe what you disliked about the recommendations you received? (Please provide a free text response.)

___________________

**Section 6: Results of this study**

You have a right to receive feedback about the overall results of this study. This feedback will be a one-page summary regarding the overall findings of the study.

Please feel free to contact Priti Kharel if you would like to receive overall feedback on the results of this study.

Priti Kharel, PhD Candidate, email: priti.kharel@sydney.edu.au phone: 02 8627 6782

**Group 3 will not receive any Choosing Wisely recommendations and go directly to the three clinical vignettes**

You will now be shown three hypothetical vignettes of patients with low back pain visiting a physiotherapist. We want you to imagine you are the physiotherapist responsible for treating these patients and indicate what test and treatment options you would consider at any point in the patient's episode of care. You are free to select as many or few options as you want.

**Section 4: Clinical vignettes**

**Hypothetical vignette 1**

**History:** A 50-year-old man has been suffering from low back pain for the past 6 weeks. He comes to see you with direct access. The pain started after he helped his 25-year-old son renovate a house. He did not lift any heavy objects. The pain is continuous and radiates to the left buttock. He called in sick to work due to the pain and has still not gone back to work. He is an electrician in a hardware store. The pain has not reduced over the past 6 weeks despite the fact that he lies down regularly. He loves to play golf, but he has not tried to play golf since he developed low back pain, he believes that playing golf will exacerbate the problem. He takes paracetamol for the pain as necessary, varying from 0-5 tablets per day.

**Physical Exam:** Vital signs: blood pressure 110/70 mmHg and pulse rate 60. During range of motion testing, he experiences some pain during back extension and lateral flexion, particularly to the right (these are not noticeably limited), but flexion is nearly impossible. The straight leg raise test on the left provokes low back pain at 80 degrees. He is not willing to lift a 20-lb weight from the floor, because he expects it will further damage his back. He assesses his control over the pain as low and lacks confidence that he could control the pain. Palpation and accessory motion testing did not reproduce low back pain symptoms; however, tenderness was noted diffusely and bilaterally from L1 to L5.

Please choose from the following list of tests and treatments that you believe would be appropriate for the patient described in vignette 1

- Refer for Bone Scan
- Refer for CT
- Refer for magnetic resonance imaging
- Refer for radiographs
- Refer for ultrasound
- Refer to the medical doctor and maintain physiotherapy treatment
- Refer to the medical doctor without intervention
- Advice to maintain an upright posture during bending and lifting
- Advice to pursue or maintain an active lifestyle
- Aerobic and fitness exercises
- Back school (a booklet that includes instructions on the home exercise program)
- Bed rest
- Directional preference exercises: extension
- Directional preference exercises: flexion
- Gradual exercise exposure
- Ice or heat
- Interferential current or transcutaneous electrical nerve stimulation (TENS)
- Laser or ultrasound
- Lumbar brace or corset
- Lumbar stabilisation exercises
- Mechanical traction
- Neurodynamic mobilisation
- Pain neuroscience education (e.g. "pain does not equal damage")
- Psychologically informed physiotherapy (behavioural therapy + regular physiotherapy)
- Spinal manipulation (thrust)
- Spinal mobilisation (non-thrust)
- Other (specify) __________________________________________________

**Hypothetical vignette 2**

**History:** A 28-year-old woman has suffered from low back pain on and off for 45 days. Low back pain started insidiously and does not appear to change with physical activity or posture. She works as a schoolteacher. She sits all day. Her back pain is worse in the end of the day and at night. The pain does not radiate to the legs; it is located on the low back and left buttock area. She has been married for five years; she has no children and is trying to become pregnant. Her medical history is unremarkable, except that her period is 6 weeks late and she reports a pinkish vaginal discharge. She is taking 200 mg ibuprofen four times a day for the back pain.

**Physical exam**: Vital signs: blood pressure 90/60 mm Hg and pulse rate 102. Back range of motion is full of pain at end range of flexion. Straight leg raise stretch the hamstrings bilaterally at 80 degrees of hip flexion. The neurological exam is within normal limits. Palpation and accessory motion testing did not reproduce low back pain symptoms; however, tenderness was noted from the L3 to the L5 lumbar vertebrae with PA pressure.

Please choose from the following list of tests and treatments that you believe would be appropriate for the patient described in vignette 2

- Refer for Bone Scan
- Refer for CT
- Refer for magnetic resonance imaging
- Refer for radiographs
- Refer for ultrasound
- Refer to the medical doctor and maintain physical therapy treatment
- Refer to the medical doctor without intervention
- Advice to maintain an upright posture during bending and lifting
- Advice to pursue or maintain an active lifestyle
- Aerobic and fitness exercises
- Back school (a booklet that includes instructions on the home exercise program)
- Bed rest
- Directional preference exercises: extension
- Directional preference exercises: flexion
- Gradual exercise exposure
- Ice or heat
- Interferential current or transcutaneous electrical nerve stimulation (TENS)
- Laser or ultrasound
- Lumbar brace or corset
- Lumbar stabilisation exercises
- Mechanical traction
- Neurodynamic mobilisation
- Pain neuroscience education (e.g. "pain does not equal damage")
- Psychologically informed physiotherapy (behavioural therapy + regular physiotherapy)
- Spinal manipulation (thrust)
- Spinal mobilisation (non-thrust)
- Other (specify)___________________________________________________

**Hypothetical vignette 3**

**History:** A 55-year-old male is referred to physical therapy complaining of low back pain. He is a heavy smoker. The pain started suddenly 2 weeks ago. The patient has noticed significant weight loss; he mentioned that he dropped two sizes. Pain is constant and does not seem to be related to changes in positions or physical activities. Pain is localised at the L1- L2 level and not referring to the lower extremities. The patient reported that the pain severity peaks at midnight with excessive, unusual sweating.

**Physical examination:** Heart rate was 104 beats per minute, blood pressure 145/60 mm Hg and he had an elevated body temperature. Lumbar range of motion was within normal limits with discomfort at the end range. Accessory motion testing did not exacerbate the patient’s symptoms. Tenderness was noted at the L1-2 level.

Please choose from the following list of tests and treatments that you believe would be appropriate for the patient described in vignette 3

- Refer for Bone Scan
- Refer for CT
- Refer for magnetic resonance imaging
- Refer for radiographs
- Refer for ultrasound
- Refer to the medical doctor and maintain physical therapy treatment
- Refer to the medical doctor without intervention
- Advice to maintain an upright posture during bending and lifting
- Advice to pursue or maintain an active lifestyle
- Aerobic and fitness exercises
- Back school (a booklet that includes instructions on the home exercise program)
- Bed rest
- Directional preference exercises: extension
- Directional preference exercises: flexion
- Gradual exercise exposure
- Ice or heat
- Interferential current or transcutaneous electrical nerve stimulation (TENS)
- Laser or ultrasound
- Lumbar brace or corset
- Lumbar stabilisation exercises
- Mechanical traction
- Neurodynamic mobilisation
- Pain neuroscience education (e.g. "pain does not equal damage")
- Psychologically informed physiotherapy (behavioural therapy + regular physiotherapy)
- Spinal manipulation (thrust)
- Spinal mobilisation (non-thrust)
- Other (specify)__________________________________________________

**Section 5: Information about Choosing Wisely recommendations**

Choosing Wisely is a global campaign, launched in 2012, that aims to encourage conversations between patients and healthcare providers about low-value care. Low-value care is care that provides little-to-no-benefit, causes harm, or diverts resources from care that is evidence-based, safe and truly necessary (high-value care). Since its launch, the campaign has been endorsed by over 250 professional societies around the world, including the Australian Physiotherapy Association (APA), with each society publishing their own list of recommendations against the use of low-value tests and treatments. In 2015, the APA published 6 recommendations against the use of test and treatments that are potentially low-value and frequently provided by physiotherapists. Two of the APA recommendations are relevant to the management of low back pain:

1. **Don't request imaging for patients with non-specific low back pain and no indicators of a serious cause for low back pain.**
2. **Avoid using electrotherapy modalities in the management of patients with low back pain.**

Please rate your agreement with the following Choosing Wisely recommendations for physiotherapists.

|  | Strongly agree | Somewhat  agree | Neither agree nor disagree | Somewhat  disagree | Strongly disagree |
| --- | --- | --- | --- | --- | --- |
| **Don’t request imaging for patients with non-specific low back pain and no indicators of a serious cause for low back pain** |  |  |  |  |  |
| **Avoid using electrotherapy modalities in the management of patients with low back pain** |  |  |  |  |  |

1. How familiar were you with the Australia Physiotherapy Association’s Choosing Wisely recommendations prior to being invited to complete this survey? (For the groups not shown the recommendations, go directly to section 6 after this question)

- Extremely familiar
- Very familiar
- Moderately familiar
- Slightly familiar
- Not familiar at all

**Section 6: Results of this study**

You have a right to receive feedback about the overall results of this study. This feedback will be a one-page summary regarding the overall findings of the study.

Please feel free to contact Priti Kharel if you would like to receive overall feedback on the results of this study.

Priti Kharel, PhD Candidate, email: priti.kharel@sydney.edu.au phone: 02 8627 6782

**Appendix 6: Test/Treatment Choices Categorisation**

| Broad categories | Test/treatment choices |
| --- | --- |
| Refer to doctor | Refer to the medical doctor and maintain physiotherapy treatment |
|  | Refer to the medical doctor without intervention |
| Advice and education | Advice to maintain an upright posture during bending and lifting |
|  | Advice to pursue or maintain an active lifestyle |
|  | Pain neuroscience education (e.g. "pain does not equal damage") |
|  | Psychologically informed physiotherapy (behavioural therapy in addition to usual care physiotherapy) |
| Exercise | Aerobic and fitness exercises |
|  | Back school (a booklet that includes instructions on a home exercise program) |
|  | Directional preference exercises: extension |
|  | Directional preference exercises: flexion |
|  | Gradual exercise exposure |
|  | Lumbar stabilisation exercises |
| Heat, cold and bracing | Ice or heat |
|  | Lumbar brace or corset |
| Bed rest | Bed rest |
| Manual therapy | Mechanical traction |
|  | Neurodynamic mobilisation |
|  | Spinal manipulation (thrust) |
|  | Spinal mobilisation (non-thrust) |
| Other | Cupping, massage and taping, relaxation, soft tissue mobilisation, exploring medication efficiency |

**Appendix 7: Comparison of demographic characteristics between participants who completed the survey and those who consented to participate but left before the randomisation**

| Characteristics | Included n (%) | Excluded n (%) |
| --- | --- | --- |
| Age (years) | **n=473** | **n=58** |
| <20 | 2 (1%) | 0 |
| 20-29 | 77 (16%) | 13 (22%) |
| 30-39 | 171 (36%) | 21 (36%) |
| 40-49 | 170 (36%) | 17 (29%) |
| 50+ | 53 (11%) | 7 (12%) |
| Sex | **n=473** | **n=58** |
| Male | 280 (59%) | 31 (53%) |
| Female | 193 (41%) | 25 (43%) |
| Non-binary/third gender | 0 | 1 (2%) |
| Prefer not to say | 0 | 1 (2%) |
| Number of LBP patients seen in last 12 months | **n=473** | **n=58** |
| 1 to 5 | 22 (5%) | 8 (14%) |
| 6 to 50 | 203 (43%) | 29 (50%) |
| 51 to 100 | 158 (33%) | 12 (21%) |
| >100 | 90 (19%) | 9 (15%) |
| Qualification* | **n=473** | **n=36** |
| Bachelors | 210 (44%) | 14 (39%) |
| Masters | 228 (48%) | 11 (31%) |
| PhD | 55 (12%) | 10 (28%) |
| Titling | 29 (6%) | 1 (3%) |
| Specialisation | 172 (36%) | 9 (25%) |
| Other qualification | 19 (4%) | 3 (8%) |
| Country of practice | **n=473** | **n=36** |
| United States | 164 (35%) | 3 (19%) |
| United Kingdom | 138 (29%) | 2 (6%) |
| Australia | 93 (20%) | 7 (3%) |
| Ireland | 9 (2%) | 2 (6%) |
| Brazil | 3 (0.6%) | 2 (6%) |
| Canada | 2 (0.42%) | 1 (8%) |
| Others | 64 (14 %) | 19 (53%) |
| Years of experience | **n=473** | **n=36** |
| <1 year | 17 (4%) | 1 (3%) |
| 1-5 years | 92 (20%) | 6 (17%) |
| 6-10 years | 156 (33%) | 8 (22%) |
| 10+ | 208 (44%) | 21 (58%) |
| Clinical area of interest* | **n=473** | **n=36** |
| Musculoskeletal | 398 (84%) | 26 (72%) |
| Sports | 208 (44%) | 11 (31%) |
| Orthopaedics | 182 (38%) | 16 (44%) |
| Cardiothoracic | 137 (29%) | 1 (3%) |
| Neurology | 132 (28%) | 5 (14%) |
| Ergonomics and occupational health | 63 (13%) | 2 (6%) |
| Continence and women's health | 45 (10%) | 4 (11%) |
| Gerontology | 44 (9%) | 2 (6%) |
| Paediatrics | 35 (7%) | 2 (6%) |
| Oncology | 28 (6%) | 2 (6%) |
| Others | 15 (3%) | 2 (6%) |
| Settings currently practising* | **n=473** | **n=36** |
| Private practice | 228 (48%) | 18 (50%) |
| Public hospital | 171 (36%) | 13 (36%) |
| Private hospital | 110 (23%) | 0 (0%) |
| Sports teams | 48 (10%) | 3 (8%) |
| Aged care | 31 (7%) | 1 (3%) |
| Others | 20 (4%) | 6 (17%) |
| Involvement in other professional activities* | **n=473** | **n=36** |
| Teaching physiotherapy students | 157 (33%) | 15 (42%) |
| Research | 113 (24%) | 17 (47%) |
| Teaching continuing education courses | 92 (119%) | 10 (28%) |
| Others | 6 (1%) | 1 (3%) |
| None | 234 (49%) | 13 (36%) |

*Multiple responses selected

n: number of participants in each group

**Appendix 8. Effect of Choosing Wisely recommendations on physiotherapists’ intentions to use imaging and electrotherapy for low back pain compared to no recommendation (excluding poor quality responses)**

|  | Original | Optimised | No recommendation | Recommendation vs no recommendation (OR, 95% CI) | Optimised vs original (OR, 95% CI) |
| --- | --- | --- | --- | --- | --- |
| Vignette 1 (N= 325) | **n=106** | **n=112** | **n=107** |  |  |
| Imaging | 63 (59%) | 58 (51%) | 58 (54%) | 1.0 (0.6 to 1.6) | 0.8 (0.4 to 1.3) |
| Electrotherapy | 14 (13%) | 13 (12%) | 16 (15%) | 0.8 (0.4 to 1.6) | 0.9 (0.4 to 1.2) |
| Vignette 2 (N= 325) | **n=106** | **n=112** | **n=107** |  |  |
| Imaging | 59 (56%) | 63 (56%) | 59 (55%) | 0.9 (0.6 to 1.6) | 1.1 (0.6 to 1.9) |
| Electrotherapy | 16 (15%) | 14 (13%) | 13 (12%) | 1.2 (0.6 to 2.3) | 0.9 (0.4 to 1.9) |
| Vignette 3 (N= 314) | **n=105** | **n=102** | **n=107** |  |  |
| Imaging | 66 (62%) | 69 (62%) | 75 (70%) | 0.6 (0.4 to 1.1) | 1.0 (0.6 to 1.7) |
| Electrotherapy | 18 (17%) | 16 (14%) | 14 (13%) | 1.3 (0.6 to 2.4) | 0.9 (0.4 to 1.8) |

N: number of participants in each vignette; n: number of participants in each group; OR: Odds Ratio; 95% CI: 95% Confidence Interval

**Appendix 9: Frequency and proportion of imaging and electrotherapy modalities chosen by participants in each of the three vignettes**

|  | Original | Optimised | No recommendation | Total |
| --- | --- | --- | --- | --- |
| Vignette 1 (N= 325) |  |  |  |  |
| Imaging responses |  |  |  |  |
| Refer for Bone Scan | 22 (3%) | 17 (2%) | 15 (2%) | 54 (2%) |
| Refer for CT | 39 (5%) | 34 (5%) | 42 (5%) | 115 (5%) |
| Refer for magnetic resonance imaging | 37 (5%) | 35 (5%) | 36 (5%) | 108 (5%) |
| Refer for radiographs | 14 (2%) | 14 (2%) | 16 (2%) | 44 (2%) |
| Refer for ultrasound | 23 (3%) | 28 (4%) | 23 (3%) | 74 (3%) |
| Electrotherapy responses |  |  |  |  |
| Interferential current or transcutaneous electrical nerve stimulation (TENS) | 10 (1%) | 12 (2%) | 15 (2%) | 37 (2%) |
| Laser or ultrasound | 15 (2%) | 11 (1%) | 9 (1%) | 35 (2%) |
|  |  |  |  |  |
| Vignette 2 (N= 325) |  |  |  |  |
| Imaging responses |  |  |  |  |
| Refer for Bone Scan | 10 (2%) | 15 (2%) | 13 (2%) | 38 (2%) |
| Refer for CT | 32 (5%) | 34 (6%) | 33 (5%) | 99 (5%) |
| Refer for magnetic resonance imaging | 34 (6%) | 38 (6%) | 41 (6%) | 113 (6%) |
| Refer for radiographs | 17 (3%) | 22 (4%) | 18 (3%) | 57 (3%) |
| Refer for ultrasound | 30 (5%) | 44 (7%) | 27 (4%) | 101 (5%) |
| Electrotherapy responses |  |  |  |  |
| Interferential current or transcutaneous electrical nerve stimulation (TENS) | 8 (1%) | 9 (1%) | 11 (2%) | 28 (1%) |
| Laser or ultrasound | 14 (2%) | 16 (3%) | 13 (2%) | 43 (2%) |
|  |  |  |  |  |
| Vignette 3 (N= 314) |  |  |  |  |
| Imaging responses |  |  |  |  |
| Refer for Bone Scan | 17 (3%) | 14 (3%) | 19 (3%) | 50 (3%) |
| Refer for CT | 37 (7%) | 42 (8%) | 43 (7%) | 122 (7%) |
| Refer for magnetic resonance imaging | 44 (8%) | 49 (10%) | 56 (9%) | 149 (9%) |
| Refer for radiographs | 30 (5%) | 28 (6%) | 26 (4%) | 84 (5%) |
| Refer for ultrasound | 25 (4%) | 36 (7%) | 23 (4%) | 84 (5%) |
| Electrotherapy responses |  |  |  |  |
| Interferential current or transcutaneous electrical nerve stimulation (TENS) | 8 (1%) | 6 (1%) | 12 (2%) | 26 (2%) |
| Laser or ultrasound | 21 (4%) | 18 (4%) | 11 (2%) | 50 (3%) |

N: total number of responses in each vignette,

**Appendix 10. Physiotherapists’ intentions to provide other treatments for low-back pain**

|  | Original | Optimised | No recommendation | Recommendation vs no recommendation (OR, 95% CI) | Optimised vs original (OR, 95% CI)* |
| --- | --- | --- | --- | --- | --- |
| Vignette 1 (N=473) | **n=150** | **n=165** | **n=158** |  |  |
| Refer to doctor | **29 (19%)** | **46 (28%)** | **39 (25%)** | **0.9 (0.6 to 1.5)** | **1.6 (0.9 to 2.7)** |
| Refer to the medical doctor and maintain physiotherapy treatment | 21 (14%) | 34 (21%) | 28 (18%) | 0.9 (0.6 to 1.6) | 1.6 (0.9 to 2.9) |
| Refer to the medical doctor without intervention | 12 (8%) | 13 (8%) | 15 (9%) | 0.8 (0.4 to 1.7) | 1.0 (0.4 to 2.3) |
| Advice and education | **83 (55%)** | **86 (52%)** | **88 (56%)** | **0.9 (0.6 to 1.3)** | **0.8 (0.6 to 1.4)** |
| Advice to maintain an upright posture during bending and lifting | 21 (14%) | 26 (16%) | 24 (15%) | 1.1 (0.6 to 1.7) | 1.2 (0.6 to 2.1) |
| Advice to pursue or maintain an active lifestyle | 45 (30%) | 53 (32%) | 45 (28%) | 1.1 (0.7 to 1.7) | 1.1 (0.7 to 1.8) |
| Pain neuroscience education (e.g. "pain does not equal damage") | 50 (33%) | 50 (30%) | 59 (37%) | 0.8 (0.5 to 1.2) | 0.9 (0.5 to 1.4) |
| Psychologically informed physiotherapy (behavioural therapy in addition to usual care physiotherapy) | 43 (29%) | 33 (20%) | 42 (27%) | 0.9 (0.6 to 1.4) | 0.6 (0.4 to 1.0) |
| Exercise | **103 (69%)** | **100 (61%)** | **110 (70%)** | **0.8 (0.5 to 1.2)** | **0.7 (0.4 to 1.1)** |
| Aerobic and fitness exercises | 35 (23%) | 44 (27%) | 43 (27%) | 0.9 (0.6 to 1.4) | 1.2 (0.7 to 2.0) |
| Back school (a booklet that includes instructions on a home exercise program) | 17 (11%) | 25 (15%) | 17 (11%) | 1.3 (0.7 to 2.4) | 1.4 (0.7 to 2.8) |
| Directional preference exercises: extension | 27 (18%) | 20 (12%) | 30 (19%) | 0.8 (0.5 to 1.2) | 0.6 (0.3 to 1.1) |
| Directional preference exercises: flexion | 23 (15%) | 25 (15%) | 32 (20%) | 0.7 (0.4 to 1.1) | 1.0 (0.5 to 1.8) |
| Gradual exercise exposure | 66 (44%) | 68 (41%) | 65 (41%) | 1.0 (0.7 to 1.6) | 0.9 (0.6 to 1.4) |
| Lumbar stabilisation exercises | 24 (16%) | 33 (20%) | 37 (23%) | 0.7 (0.5 to 1.2) | 1.3 (0.7 to 2.4) |
| Heat, cold and bracing | **67 (45%)** | **71 (43%)** | **81 (51%)** | **0.8 (0.5 to 1.1)** | **0.9 (0.6 to 1.5)** |
| Ice or heat | 61 (41%) | 61 (37%) | 72 (46%) | 0.8 (0.5 to 1.1) | 0.9 (0.6 to 1.4) |
| Lumbar brace or corset | 11 (7%) | 14 (8%) | 16 (10%) | 0.8 (0.4 to 1.5) | 1.2 (0.5 to 2.7) |
| Bed rest | **46 (31%)** | **40 (24%)** | **44 (28%)** | **1.0 (0.6 to 1.6)** | **0.8 (0.4 to 1.2)** |
| Manual therapy | **44 (29%)** | **42 (26%)** | **47 (30%)** | **0.9 (0.6 to 1.3)** | **0.8 (0.5 to 1.4)** |
| Mechanical traction | 8 (5%) | 10 (6%) | 10 (6%) | 0.9 (0.4 to 1.9) | 1.1 (0.4 to 3.0) |
| Neurodynamic mobilisation | 20 (13%) | 20 (12%) | 23 (15%) | 0.9 (0.5 to 1.5) | 0.9 (0.5 to 1.8) |
| Spinal manipulation (thrust) | 8 (5%) | 7 (4%) | 9 (6%) | 0.8 (0.3 to 1.8) | 0.7 (0.3 to 2.1) |
| Spinal mobilisation (non-thrust) | 21 (14%) | 18 (11%) | 20 (13%) | 1.0 (0.5 to 1.7) | 0.7 (0.4 to 1.5) |
| Other (e.g. cupping, massage and taping, relaxation, soft tissue mobilisation, explore medication efficiency) | **5 (1%)** | **9 (1%)** | **9 (1%)** | **0.8 (0.3 to 1.8)** | **1.6 (0.5 to 5.1)** |
|  |  |  |  |  |  |
| Vignette 2 (N=467) | **n=150** | **n=165** | **n=158** |  |  |
| Refer to doctor | **67 (45%)** | **90 (55%)** | **75 (48%)** | **1.1 (0.8 to 1.6)** | **1.4 (0.9 to 2.3)** |
| Refer to the medical doctor and maintain physiotherapy treatment | 38 (25%) | 54 (33%) | 39 (25%) | 1.2 (0.8 to 1.9) | 1.4 (0.9 to 2.3) |
| Refer to the medical doctor without intervention | 33 (22%) | 39 (24%) | 38 (24%) | 0.9 (0.6 to 1.5) | 1.1 (0.7 to 1.9) |
| Advice and education | **71 (47%)** | **66 (40%)** | **70 (44%)** | **1.0 (0.7 to 1.4)** | **0.7 (0.5 to 1.2)** |
| Advice to maintain an upright posture during bending and lifting | 19 (13%) | 24 (15%) | 23 (15%) | 0.9 (0.5 to 1.6) | 1.2 (0.6 to 2.2) |
| Advice to pursue or maintain an active lifestyle | 36 (24%) | 35 (21%) | 39 (25%) | 0.9 (0.6 to 1.4) | 0.9 (0.5 to 1.5) |
| Pain neuroscience education (e.g. "pain does not equal damage") | 37 (25%) | 22 (13%) | 30 (19%) | 1.0 (0.6 to 1.6) | 0.5 (0.3 to 0.8) |
| Psychologically informed physiotherapy (behavioural therapy in addition to+ regular usual care physiotherapy) | 37 (25%) | 21 (13%) | 35 (22%) | 0.8 (0.5 to 1.3) | 0.5 (0.2 to 0.8) |
| Exercise | **76 (51%)** | **77 (47%)** | **88 (56%)** | **0.8 (0.5 to 1.1)** | **0.9 (0.5 to 1.3)** |
| Aerobic and fitness exercises | 34 (23%) | 25 (15%) | 36 (23%) | 0.8 (0.5 to 1.3) | 0.6 (0.3 to 1.1) |
| Back school (a booklet that includes instructions on a home exercise program) | 16 (11%) | 12 (7%) | 20 (13%) | 0.7 (0.4 to 1.3) | 0.7 (0.3 to 1.5) |
| Directional preference exercises: extension | 21 (14%) | 3 (2%) | 11 (7%) | 1.1 (0.5 to 2.3) | 0.1 (0.0 to 0.4) |
| Directional preference exercises: flexion | 15 (10%) | 8 (5%) | 20 (13%) | 0.6 (0.3 to 1.0) | 0.5 (0.2 to 1.1) |
| Gradual exercise exposure | 26 (17%) | 37 (22%) | 34 (22%) | 0.9 (0.6 to 1.5) | 1.4 (0.8 to 2.4) |
| Lumbar stabilisation exercises | 25 (17%) | 33 (20%) | 32 (20%) | 0.9 (0.5 to 1.4) | 1.2 (0.7 to 2.2) |
| Heat, cold and bracing | **56 (37%)** | **43 (26%)** | **52 (33%)** | **0.9 (0.6 to 1.4)** | ***0.6 (0.4 to 0.97*** |
| Ice or heat | 46 (31%) | 37 (22%) | 43 (27%) | 1.0 (0.6 to 1.5) | 0.7 (0.4 to 1.1) |
| Lumbar brace or corset | 15 (10%) | 7 (4%) | 14 (9%) | 0.8 (0.4 to 1.5) | 0.4 (0.2 to 1.0) |
| Bed rest | **47 (31%)** | **34 (21%)** | **33 (21%)** | **1.4 (0.9 to 2.2)** | **1.0 (0.6 to 1.7)** |
| Manual therapy | **37 (25%)** | **23 (14%)** | **36 (23%)** | **0.8 (0.5 to 1.2)** | ***0.5 (0.3 to 0.9)*** |
| Mechanical traction | 5 (3%) | 7 (4%) | 10 (6%) | 0.6 (0.2 to 1.3) | 1.3 (0.4 to 4.1) |
| Neurodynamic mobilisation | 8 (5%) | 6 (4%) | 9 (6%) | 0.7 (0.3 to 1.7) | 0.6 (0.2 to 1.9) |
| Spinal manipulation (thrust) | 12 (8%) | 7 (4%) | 15 (9%) | 0.6 (0.3 to 1.2) | 0.5 (0.2 to 1.2) |
| Spinal mobilisation (non-thrust) | 17 (11%) | 9 (5%) | 12 (8%) | 1.1 (0.5 to 2.2) | 0.5 (0.2 to 1.0) |
| Other (pregnancy test, wet cupping therapy, drug therapy, refer to gynaecologist, visceral testing) | **3 (0.5%)** | **12 (2%)** | **10 (2%)** | **0.7 (0.3 to 1.6)** | **3.8 (1.0 to 13.7)** |
|  |  |  |  |  |  |
| Vignette 3 (N=449) | **n=150** | **n=165** | **n=158** |  |  |
| Refer to doctor | **77 (51%)** | **73 (44%)** | **83 (52%)** | **0.8 (0.6 to 1.2)** | **0.8 (0.5 to 1.2)** |
| Refer to the medical doctor and maintain physiotherapy treatment | 25 (17%) | 22 (13%) | 32 (20%) | 0.7 (0.4 to 1.1) | 0.7 (0.4 to 1.4) |
| Refer to the medical doctor without intervention | 57 (38%) | 54 (33%) | 55 (35%) | 1.0 (0.7 to 1.5) | 0.8 (0.5 to 1.3) |
| Advice and education | **49 (33%)** | **46 (28%)** | **65 (41%)** | ***0.6 (0.4 to 0.9)*** | **0.8 (0.5 to 1.3)** |
| Advice to maintain an upright posture during bending and lifting | 12 (8%) | 12 (7%) | 22 (14%) | *0.5 (0.3 to 0.9)* | 0.9 (0.4 to 2.0) |
| Advice to pursue or maintain an active lifestyle | 19 (13%) | 19 (12%) | 33 (21%) | *0.5 (0.3 to 0.9)* | 0.9 (0.5 to 1.8) |
| Pain neuroscience education (e.g. "pain does not equal damage") | 20 (13%) | 17 (10%) | 27 (17%) | 0.7 (0.4 to 1.1) | 0.8 (0.4 to 1.5) |
| Psychologically informed physiotherapy (behavioural therapy in addition to+ regular usual care physiotherapy) | 26 (17%) | 19 (12%) | 28 (18%) | 0.8 (0.5 to 1.3) | 0.6 (0.3 to 1.2) |
| Exercise | **57 (38%)** | **51 (31%)** | **64 (41%)** | **0.8 (0.5 to 1.1)** | **0.7 (0.5 to 1.2)** |
| Aerobic and fitness exercises | 19 (13%) | 12 (7%) | 26 (16%) | 0.6 (0.3 to 1.0) | 0.5 (0.3 to 1.1) |
| Back school (a booklet that includes instructions on a home exercise program) | 6 (4%) | 7 (4%) | 11 (7%) | 0.6 (0.3 to 1.4) | 1.1 (0.4 to 3.4) |
| Directional preference exercises: extension | 15 (10%) | 3 (2%) | 12 (8%) | 0.8 (0.4 to 1.6) | 0.2 (0.1 to 0.6) |
| Directional preference exercises: flexion | 10 (7%) | 6 (4%) | 9 (6%) | 0.9 (0.4 to 2.1) | 0.5 (0.2 to 1.5) |
| Gradual exercise exposure | 20 (13%) | 24 (15%) | 19 (12%) | 1.2 (0.7 to 2.0) | 1.1 (0.6 to 2.0) |
| Lumbar stabilisation exercises | 20 (13%) | 18 (11%) | 25 (16%) | 0.7 (0.4 to 1.3) | 0.8 (0.4 to 1.6) |
| Heat, cold and bracing | **52 (35%)** | **40 (24%)** | **47 (30%)** | **1.0 (0.7 to 1.6)** | **0.6 (0.4 to 1.0)** |
| Ice or heat | 42 (28%) | 30 (18%) | 40 (25%) | 0.9 (0.6 to 1.4) | 0.6 (0.3 to 1.0) |
| Lumbar brace or corset | 16 (11%) | 11 (7%) | 12 (8%) | 1.1 (0.6to 2.3) | 0.6 (0.3 to 1.3) |
| Bed rest | **47 (31%)** | **34 (21%)** | **33 (21%)** | **1.4 (0.9 to 2.1)** | **0.7 (0.4 to 1.2)** |
| Manual therapy | **23 (15%)** | **21 (13%)** | **27 (17%)** | **0.8 (0.5 to 1.3)** | **0.8 (0.4 to 1.5)** |
| Mechanical traction | 9 (6%) | 4 (2%) | 8 (5%) | 0.8 (0.3 to 2.0) | 0.4 (0.1 to 1.3) |
| Neurodynamic mobilisation | 6 (4%) | 6 (4%) | 11 (7%) | 0.5 (0.2 to 1.2) | 0.9 (0.3 to 2.8) |
| Spinal manipulation (thrust) | 8 (5%) | 12 (7%) | 13 (8%) | 0.7 (0.3 to 1.5) | 1.3 (0.5 to 3.4) |
| Spinal mobilisation (non-thrust) | 5 (3%) | 2 (1%) | 2 (1%) | 1.7 (0.3 to 8.2) | 0.3 (0.1 to 1.8) |
| Other (Emergency Department referral for possible malignancy, reassurance, blood tests) | **4 (1%)** | **4 (1%)** | **6 (1%)** | **0.6 (0.2 to 1.9)** | **0.9 (0.2 to 3.6)** |

N: number of participants in each vignette; n: number of participants in each group; OR: Odds Ratio; 95% CI: 95% Confidence Interval

Italicised text means p<0.05
