# Supplementary material for: Effectiveness of Choosing Wisely recommendations in reducing physiotherapists’ intentions to refer for imaging and use electrotherapy for low back pain: a randomised controlled experiment
Source: BMJ Open. 2025 Jun 26;15(6):e097202. doi: 10.1136/bmjopen-2024-097202 (PMC12207150; doi:10.1136/bmjopen-2024-097202)
Supplement: Supplementary file 2 [file bmjopen-2024-097202.reviewer_comments.pdf]

PEER REVIEW HISTORY

BMJ Open publishes all reviews undertaken for accepted manuscripts. Reviewers are asked to complete a checklist review form and are provided with free text boxes to elaborate on their assessment. These free text comments are reproduced below.

ARTICLE DETAILS

Title (Provisional)

Effectiveness of Choosing Wisely recommendations in reducing physiotherapists' intentions to refer for imaging and use electrotherapy for low back pain: a randomised controlled experiment

Authors

Kharel, Priti; Maher, Chris; Gamble, Andrew R; Ferreira, Giovanni E; Zadro, Joshua

---

VERSION 1 - REVIEW

**Reviewer**	**1**
**Name**	**Gleadhill, Connor**
**Affiliation**	**The University of Newcastle, School of Medicine and Public Health**
**Date**	**06-Feb-2025**
**COI**	**None**

I congratulate the authoring team on an important study. The study is a good addition to the field and is worthy of publication. Some changes would strengthen the publication, notably in presentation of outcome measures, results, and certain discussion points. There is some conflation in the discussion around behaviour and intention to refer or use a therapy; it is important to be specific around what you can sensibly interpret from the study - only intention.

METHOD

Section 2.2:

1. Recommend removing the sentence beginning 'The market research panels..' as it is duplicative.

Section 2.6:

2. Recommend that you provide a table to better describe the outcome measures. Including variable categories, structure, values.

Section 2.7

3. Recommend providing evidence of the detectable effect size - i.e., what evidence is behind the 10% between group difference.

Section 2.8:

4. Recommend specifying that you did not employ intention-to-treat analyses.

RESULTS:

General comment:

5. It would be worthwhile to provide an appendix with the frequency and proportion of the imaging and electrotherapy modality that were chosen in each of the vignettes. This would be interesting for the more clinically-minded reader.

Table 1:

6. The number of low back pain patients treated in the prior 12 months from screening questions is useful information. Recommend including it in participant characteristics table.

Section 3.3 Primary outcomes

7. Sentence starting, "The use of electrotherapy was not a major issue.." pg 13, line 30. Suggest re-wording, simply state the intention to use electrotherapy was low across all vignettes (provide range for vignettes).

8. Sentence starting, "However, imaging use was found.." pg 13, line31. Recommend removing this as it is an interpretation of results and about use (not intention to use). Bringing the information about appropriateness of imaging and electrotherapy will let the reader interpret. To make this kind of statement (about understanding of guidelines) a different study is likely needed.

9. Table 2: Bring the information about appropriateness of imaging and electrotherapy into the table for easier interpretation. The table labels is also incorrect - imaging inappropriate for vignettes 1 & 2, but appropriate for 3.

DISCUSSION

General comment

10. Some discussion about the participant characteristics may be important. For example, the country in which someone practices may shape their intentions to refer for imaging because of different referral legislation and cultural practices.

Also, the frequency with which clinicians see patients with low back pain, and their experience in managing these patients are both clinically interesting points that the study could discuss.

11. Paragraph four, pg 16 line 31: Some duplication of this paragraph with paragraphs four and five. Recommend condensing into one paragraph that has a more nuanced discussion on the study evaluating the effects of CW, and the wording of CW recommendations, on

intention to refer for imaging or provide electrotherapy. Intentions are an important determinant of behaviour. Behavioural context is an important determinant to consider in implementation strategies (with appropriate references to implementation frameworks).

12. page 16, line 36: Please change "physiotherapists' treatment decisions" to "intentions about treatment decisions".

13. Page 17, line 35: Please change "chose to refer" to "reported that they intend to refer for imaging or electrotherapy."

14. Page 17, line 38: Sentence at beginning of sixth paragraph. While this may be the case, this statement is not accompanied by any references. Recommend removing, as the current study cannot support this kind of interpretation.

15. General comment on sixth paragraph: This paragraph is exclusively about behaviour. Recommend removing this interpretation.

The final two sentences in this paragraph can go into a more nuanced discussion about intention being a component of behaviour. To modify behaviour, it is important to consider contextual factors.

The reviewer provided a marked copy with additional comments. Please contact the publisher for full details.

**Reviewer**	**2**
**Name**	**Gibbs, Mitchell T.**
**Affiliation**	**University of New South Wales**
**Date**	**04-Mar-2025**
**COI**	**None**

Thank you for the opportunity to review this paper. This was a randomised survey design exploring the choosing wisely recommendations in their original form and in an optimised version. The study design is excellent and I applaud the authors using a randomised design in this form of work, which is further strengthened by the large sample. Below, I present some considerations for the authors.

I am curious why the authors did not further characterize the sample with something like the PABS-PT (https://pubmed.ncbi.nlm.nih.gov/15737810/), which is common in this field of research. The survey appears to have asked for self-reported perceptions of knowledge on these topics, which may be influenced by treatment orientation (e.g., a biomedical orientation compared to a biopsychosocial one when both rate ‘extremely knowledgeable’ may look like a very different answer…?) the effect of BM/BPS orientation has been shown regarding decision-making https://pubmed.ncbi.nlm.nih.gov/32947498/

https://pubmed.ncbi.nlm.nih.gov/37506584/ and treatment confidence/utilisation https://pubmed.ncbi.nlm.nih.gov/38054520/)

This was a large, global sample. Given the heterogenous contexts physiotherapists work in globally, what influence might this have on the outcome compared to if this was explored say only in the US or similar?

Was the study and analysis pre-protocolised somewhere such as OSF?

Optimised recommendations: I am curious why the authors did not further 'optimise' this? Why not give the red flags and talk in absence of cluster (as done well in this paper when talking about the role of assessment in exercise-based practice: https://pubmed.ncbi.nlm.nih.gov/34676659/) for imaging-related items? This is not clear in this paper alone and seems the authors should expand more on their decision-making around optimisation in this paper.

28% of the sample had a special interest in oncology? Did the authors specify this was related to non-cancer pain? Could this bias the result in this sample as a previous history of cancer is considered a red flag?

Discussion: the discussion is generally well written. I do feel there is a bit of a need for the reader to go and read the best-worst scaling study and qualitative study to completely grasp the authors points. As these (especially the best-worst scaling) appear to be quite linked to this study, I would suggest the authors provide some more key information to the reader.

VERSION 1 - AUTHOR RESPONSE

<u>REVIEWER 1</u>
COMMENTS TO THE AUTHORS

I congratulate the authoring team on an important study. The study is a good addition to the field and is worthy of publication. Some changes would strengthen the publication, notably in presentation of outcome measures, results, and certain discussion points. There is some conflation in the discussion around behaviour and intention to refer or use a therapy; it is important to be specific around what you can sensibly interpret from the study - only intention.

AUTHORS' RESPONSE
We appreciate the reviewer's feedback on our review. We acknowledge that our study measured intentions, not actual clinical behaviour. We have now replaced any mention of behaviour change with change in intention throughout the discussion.

COMMENTS TO THE AUTHORS
METHOD
Section 2.2:

Recommend removing the sentence beginning 'The market research panels..' as it is duplicative.

AUTHORS' RESPONSE
Thank you for your feedback. We have now removed the sentence.

(Page 6, 4th paragraph)

> We recruited registered and practising physiotherapists who had treated patients with low back pain in the past 12 months. We did not restrict participants based on their age (provided they were 18 years or older), sex, clinical experience, area of speciality, or country of practice. We recruited participants via social media (e.g. Facebook, X-formerly Twitter) and through the market research company, Qualtrics. Qualtrics recruits eligible participants from over 20 panel providers globally. Most participants come from traditional, actively managed market research panels, although social media is occasionally used for recruitment purposes. Qualtrics utilises random sampling methods and compensates participants by offering incentives such as cash, airline miles, or gift cards, uses advanced digital fingerprinting technology and verifies each IP address to ensure the validity of responses and prevent duplication duplication[1]. ~~The market research panels that assist in participant recruitment for Qualtrics also utilise deduplication technology to maintain the accuracy and authenticity of survey data[1].~~

COMMENTS TO THE AUTHORS
Section 2.6:
Recommend that you provide a table to better describe the outcome measures. Including variable categories, structure, values.

AUTHORS' RESPONSE
We thank the reviewer for their feedback. We have now provided the outcome measures in a table as below:

(Page 9, 2nd paragraph)
2.6 Outcome measures
Outcomes were collected immediately after participants read each vignette. Our outcome measures are listed below with their variable categories, scale and response options (Table 1).

Table 1: Outcome measures

Outcome Measures	Variable Categories	Response scale	Response Options*
**Primary Outcomes**			
Intentions to refer for imaging	Categorical	Multiple choice	• Refer for bone scan,<br>• Refer for CT scan,<br>• Refer for MRI,

			• Refer for radiographs, and<br>• Refer for ultrasound
Intentions to use electrotherapy	Categorical	Multiple choice	• Interferential current or transcutaneous electrical nerve stimulation (TENS)<br>• Laser or ultrasound
**Secondary Outcomes**			
Intentions to provide other treatments	Categorical	Multiple choice	• Refer to doctor<br>• Refer to the medical doctor and maintain physiotherapy treatment<br>• Refer to the medical doctor without intervention<br>• Advice and education<br>• Advice to maintain an upright posture during bending and lifting<br>• Advice to pursue or maintain an active lifestyle<br>• Pain neuroscience education (e.g. "pain does not equal damage")<br>• Psychologically informed physiotherapy (behavioural therapy in addition to usual care physiotherapy)<br>• Exercise<br>• Aerobic and fitness exercises<br>• Back school (a booklet that includes instructions on a home exercise program)<br>• Directional preference exercises: extension<br>• Directional preference exercises: flexion<br>• Gradual exercise exposure<br>• Lumbar stabilisation exercises<br>• Heat, cold and bracing<br>• Ice or heat<br>• Lumbar brace or corset<br>• Bed rest<br>• Manual therapy<br>• Mechanical traction<br>• Neurodynamic mobilisation<br>• Spinal manipulation (thrust)<br>• Spinal mobilisation (non-thrust)<br>• Other (e.g. cupping, massage and taping, relaxation, soft tissue mobilisation, explore medication efficiency)
Influence of Choosing Wisely recommendations on decision-making in the vignettes	Ordinal	5-point Likert	1. A great deal,<br>2. A fair amount,<br>3. Somewhat,<br>4. Slightly,<br>5. Not at all

Familiarity with Choosing Wisely recommendations	Ordinal	5-point Likert	1. Extremely familiar,<br>2. Very familiar,<br>3. Moderately familiar,<br>4. Slightly familiar,<br>5. Not familiar at all
Agreement with Choosing Wisely recommendations against imaging for low back pain	Ordinal	5-point Likert	1. Strongly agree,<br>2. Somewhat agree,<br>3. Neither agree nor disagree,<br>4. Somewhat disagree,<br>5. Strongly disagree
Agreement with Choosing Wisely recommendations against electrotherapy for low back pain	Ordinal	5-point Likert	1. Strongly agree,<br>2. Somewhat agree,<br>3. Neither agree nor disagree,<br>4. Somewhat disagree,<br>5. Strongly disagree

*All imaging and treatment options were included in the same question following each vignette.

COMMENTS TO THE AUTHORS

Section 2.7

Recommend providing evidence of the detectable effect size - i.e., what evidence is behind the 10% between group difference.

AUTHORS' RESPONSE

We thank the reviewer for their feedback. The 10% reduction in imaging rates was based on previous trials in the field that aim to reduce imaging use for low back pain. We have now cited two articles in our methods section to support our effect size selection. Please see below:

(Page 11, 1st paragraph)

2.7 Sample size calculation

We calculated that a sample size of 468 (156 per group) would provide 80% power to detect a 10% between-group difference for our primary comparison (Choosing Wisely recommendations vs. *no recommendation*) in the percentage of physiotherapists intending to refer for imaging or use electrotherapy for low back pain (i.e. 15% of physiotherapists intending to refer for imaging and use electrotherapy in the intervention groups and 25% in the control group). This assumes a 2-tailed alpha of 0.05 and a 15% dropout. A 10% between-group effect for our primary outcomes was selected as it is an effect size commonly used for studies investigating treatment choices for low back pain[2, 3].

COMMENTS TO THE AUTHORS

Section 2.8:

Recommend specifying that you did not employ intention-to-treat analyses.

AUTHORS' RESPONSE

We thank the reviewer for their feedback. However, we believe we conformed to ITT principles as we analysed people in the groups they were allocated to, regardless of whether they read the recommendations or not. A per-protocol analysis (i.e. not intention to treat) would have only occurred if we used knowledge of people reading the Choosing Wisely recommendations (or not) to assign them to groups for analysis. This has now been added in the manuscript. Please see below:

(Page 11, 2nd paragraph)
2.8 Data analysis
Data analyses were performed according to an intention-to-treat approach. We used descriptive statistics to summarise participants' demographic and professional characteristics. Multivariable logistic regression models were used to examine the effect of Choosing Wisely recommendations on physiotherapists' intentions to refer for imaging or use electrotherapy for low back pain in each vignette. The regression included a comparison of Choosing Wisely recommendations (both *optimised* and *original recommendations* combined) vs. *no recommendation*, followed by a comparison of *optimised* vs *original Choosing Wisely recommendations.*

COMMENTS TO THE AUTHORS
RESULTS:
General comment:
It would be worthwhile to provide an appendix with the frequency and proportion of the imaging and electrotherapy modality that were chosen in each of the vignettes. This would be interesting for the more clinically-minded reader.

AUTHORS' RESPONSE
We thank the reviewer for their feedback. We have now added Appendix 9 describing the frequency and proportion of imaging and electrotherapy response options in each of the three vignettes and has also been mentioned in section 3.4. Please see below:

Appendix 9: Frequency and proportion of imaging and electrotherapy modalities chosen by participants in each of the three vignettes

	**Original**	**Optimised**	**No recommendation**	**Total**
**Vignette 1 (N= 325)**				
**Imaging responses**				
Refer for Bone Scan	22 (3%)	17 (2%)	15 (2%)	54 (2%)
Refer for CT	39 (5%)	34 (5%)	42 (5%)	115 (5%)
Refer for magnetic resonance imaging	37 (5%)	35 (5%)	36 (5%)	108 (5%)
Refer for radiographs	14 (2%)	14 (2%)	16 (2%)	44 (2%)
Refer for ultrasound	23 (3%)	28 (4%)	23 (3%)	74 (3%)
**Electrotherapy responses**				

Interferential current or transcutaneous electrical nerve stimulation (TENS)	10 (1%)	12 (2%)	15 (2%)	37 (2%)
Laser or ultrasound	15 (2%)	11 (1%)	9 (1%)	35 (2%)
**Vignette 2 (N= 325)**				
**Imaging responses**				
Refer for Bone Scan	10 (2%)	15 (2%)	13 (2%)	38 (2%)
Refer for CT	32 (5%)	34 (6%)	33 (5%)	99 (5%)
Refer for magnetic resonance imaging	34 (6%)	38 (6%)	41 (6%)	113 (6%)
Refer for radiographs	17 (3%)	22 (4%)	18 (3%)	57 (3%)
Refer for ultrasound	30 (5%)	44 (7%)	27 (4%)	101 (5%)
**Electrotherapy responses**				
Interferential current or transcutaneous electrical nerve stimulation (TENS)	8 (1%)	9 (1%)	11 (2%)	28 (1%)
Laser or ultrasound	14 (2%)	16 (3%)	13 (2%)	43 (2%)
**Vignette 3 (N= 314)**				
**Imaging responses**				
Refer for Bone Scan	17 (3%)	14 (3%)	19 (3%)	50 (3%)
Refer for CT	37 (7%)	42 (8%)	43 (7%)	122 (7%)
Refer for magnetic resonance imaging	44 (8%)	49 (10%)	56 (9%)	149 (9%)
Refer for radiographs	30 (5%)	28 (6%)	26 (4%)	84 (5%)
Refer for ultrasound	25 (4%)	36 (7%)	23 (4%)	84 (5%)
**Electrotherapy responses**				
Interferential current or transcutaneous electrical nerve stimulation (TENS)	8 (1%)	6 (1%)	12 (2%)	26 (2%)
Laser or ultrasound	21 (4%)	18 (4%)	11 (2%)	50 (3%)

N: total number of responses in each vignette

COMMENTS TO THE AUTHORS

Table 1:

The number of low back pain patients treated in the prior 12 months from screening questions is useful information. Recommend including it in participant characteristics table.

AUTHORS' RESPONSE

We have included in Table 2. Characteristics of participants. Please see below:

Table 2. Characteristics of participants

**Characteristics (N=473)**	Original recommendation (n=150)	Optimised recommendation (n=165)	No recommendation (n=158)	Total (N=473)
**Age (years), n (%)**				
<20	0 (0%)	1 (1%)	1 (1%)	2 (1%)

20-29	25 (17%)	26 (16%)	26 (17%)	77 (16%)
30-39	46 (31%)	66 (40%)	59 (37%)	171 (36%)
40-49	57 (38%)	59 (36%)	54 (34%)	170 (36%)
50+	22 (14%)	13 (8%)	18 (11%)	53 (11%)
**Sex, n (%)**				
Male	85 (57%)	102 (62%)	93 (59%)	280 (59%)
Female	65 (43%)	63 (38%)	65 (41%)	193 (41%)
**Number of LBP patients seen in last 12 months, n (%)**				
1 to 5	4 (3%)	10 (6%)	8 (5%)	22 (5%)
6 to 50	62 (41%)	66 (40%)	75 (48%)	203 (43%)
51 to 100	53 (35%)	56 (34%)	49 (31%)	158 (33%)
>100	31 (21%)	33 (20%)	26 (16%)	90 (19%)

COMMENTS TO THE AUTHORS

Section 3.3 Primary outcomes

Sentence starting, "The use of electrotherapy was not a major issue.." pg 13, line 30. Suggest re-wording, simply state the intention to use electrotherapy was low across all vignettes (provide range for vignettes).

AUTHORS' RESPONSE

Thank you for your feedback to re-word the sentence. We have now modified the results section.

(Page 18, 1st paragraph)

3.4 Physiotherapists' intentions to refer for imaging or use electrotherapy for low back pain (Primary outcome)

Across all vignettes, no statistically significant differences were found in intentions to refer for imaging or use electrotherapy between those who received *Choosing Wisely recommendations* versus *no recommendation*, or between those who received *optimised* versus *original recommendations* (Table 3). Intentions to use electrotherapy was low across all vignettes (range 12% to 17%).

COMMENTS TO THE AUTHORS

8. Sentence starting, "However, imaging use was found.." pg 13, line31. Recommend removing this as it is an interpretation of results and about use (not intention to use). Bringing the information about appropriateness of imaging and electrotherapy will let the reader interpret. To make this kind of statement (about understanding of guidelines) a different study is likely needed.

AUTHORS' RESPONSE

Thank you for the feedback. Changes have been made:

(Page 16, 1st paragraph)

However, intentions to use imaging varied, with some physiotherapists intending to use imaging when it wasn’t indicated (Vignettes 1 and 2) and some not intending to use imaging when it was indicated (Vignette 3). We also did not find any statistically significant differences in the intentions to refer for imaging or use electrotherapy among physiotherapists when poor quality responses were excluded (Appendix 8). Appendix 9 also shows the frequency and proportion of imaging and electrotherapy modalities chosen by participants in each of the three vignettes.

COMMENTS TO THE AUTHORS
9. Table 2: Bring the information about appropriateness of imaging and electrotherapy into the table for easier interpretation. The table labels is also incorrect - imaging inappropriate for vignettes 1 & 2, but appropriate for 3.

AUTHORS’ RESPONSE
We appreciate the feedback. Following your recommendation, we have modified the table for easier interpretation of appropriateness. See below highlighted:

Table 4: Physiotherapists' intentions to refer for imaging and use electrotherapy for low back pain

	**Original**	**Optimised**	**No recommendation**	**Recommendation vs no recommendation (OR, 95% CI)**	**Optimised vs original (OR, 95% CI)**
**Vignette 1 (N= 473)**	n=150	n=165	n=158		
Imaging (inappropriate)	90 (60%)	82 (59%)	86 (54%)	0.9 (0.6 to 1.4)	0.7 (0.4 to 1.0)
Electrotherapy (inappropriate)	24 (16%)	21 (13%)	23 (15%)	1.0 (0.6 to 1.7)	0.8 (0.4 to 1.5)
**Vignette 2 (N= 467)**	n=147	n=162	n=158		
Imaging (inappropriate)	81 (54%)	91 (55%)	86 (54%)	0.9 (0.6 to 1.4)	1.0 (0.7 to 1.7)
Electrotherapy (inappropriate)	21 (14%)	23 (14%)	23 (15%)	0.9 (0.5 to 1.7)	1.0 (0.5 to 1.9)
**Vignette 3 (N= 449)**	n=146	n=147	n=156		
Imaging (appropriate)	90 (60%)	98 (59%)	106 (67%)	0.7 (0.5 to 1.0)	0.9 (0.6 to 1.6)
Electrotherapy (inappropriate)	29 (19%)	22 (13%)	23 (15%)	1.1 (0.7 to 2.0)	0.7 (0.4 to 1.2)

N: total number of participants in each vignette, OR: Odds Ratio, 95% CI: 95% Confidence Interval.

COMMENTS TO THE AUTHORS

DISCUSSION

General comment

Some discussion about the participant characteristics may be important. For example, the country in which someone practices may shape their intentions to refer for imaging because of different referral legislation and cultural practices.

Also, the frequency with which clinicians see patients with low back pain, and their experience in managing these patients are both clinically interesting points that the study could discuss.

AUTHORS' RESPONSE

Following your recommendation, we have now added a paragraph about the above characteristics in our discussion. Please see below:

(Page 20, 4th paragraph)

> An important consideration in interpreting these findings is the characteristics of the participants in our study. First, most participants had good experience managing patients with low back pain, with 43% of physiotherapists seeing 6-50 patients with low back pain in the past year and 52% seeing more than 50 in the past year. This experience may have meant physiotherapists had established routines in managing patients with low back pain and were less likely to change their practice based on Choosing Wisely recommendations[4]. In contrast, a sample of less experienced physiotherapists may have been more receptive to practice change after reading the recommendations[5]. Second, most participants were from the United States (31%), United Kingdom (29%), and Australia (24%), countries with vastly different health systems. These differences likely influence how physiotherapists interpret and apply practice recommendations, could have possibly reduced the impact of the standardised Choosing Wisely recommendations.

COMMENTS TO THE AUTHORS

11. Paragraph four, pg 16 line 31: Some duplication of this paragraph with paragraphs four and five. Recommend condensing into one paragraph that has a more nuanced discussion on the study evaluating the effects of CW, and the wording of CW recommendations, on intention to refer for imaging or provide electrotherapy. Intentions are an important determinant of behaviour. Behavioural context is an important determinant to consider in implementation strategies (with appropriate references to implementation frameworks).

AUTHORS' RESPONSE

We thank the reviewer for the feedback, and we have made the required changes as per the suggestions.

(Page 21, 2nd paragraph)

> The findings of our study suggest that while physiotherapists generally support the Choosing Wisely recommendations, merely presenting the recommendations to

physiotherapists, even with modified language to be clearer and more positive, is not sufficient to change physiotherapists' intentions about treatment decisions. Many physiotherapists in our study reported that the Choosing Wisely recommendations influenced their decision-making in the vignettes and indicated high familiarity and agreement with them. However, when they were presented with both original and modified versions of two APA Choosing Wisely recommendations targeting low back pain, no significant change was observed in their intentions to refer for imaging or use electrotherapy. This contrasts with our previous qualitative study (n=19 physiotherapists) which explored attitudes, views, and beliefs towards the APA original Choosing Wisely recommendations. This study identified that the language used in the recommendations was considered a barrier to their use[5]. Similarly, our best-Worst Scaling study involved 215 physiotherapists, assessed the effect of language on physiotherapists' willingness to follow the APA Choosing Wisely recommendations[6]. . We modified the six original recommendations based on four language characteristics (level of detail, strength- qualitied/unqualified, framing and alternatives to low-value care) to create 60 recommendations[6]. The study found that modifications to the language of the recommendations, such as providing more detail, offering alternatives to low-value care, and positively framing the recommendations, increased physiotherapists' willingness to adopt them[6].

Variations in findings across these studies may be due to differences in study methods. In the qualitative study, physiotherapists were asked to discuss barriers to using the recommendations, with the language of the recommendations identified as one of the barriers[5]. In the Best-Worst Scaling study, physiotherapists rated recommendations based on their willingness to follow them, without any additional context[6]. Meanwhile, in the current study, physiotherapists were presented with three hypothetical clinical scenarios that required more complex decision-making.

COMMENTS TO THE AUTHORS

12. page 16, line 36: Please change "physiotherapists' treatment decisions" to "intentions about treatment decisions".

AUTHORS' RESPONSE

Thank you for your suggestion. Changes have been made.

(Page 21, 2nd paragraph)

The findings of our study suggest that while physiotherapists generally support the Choosing Wisely recommendations, merely presenting the recommendations to physiotherapists, even with modified language to be clearer and more positive, is not sufficient to change physiotherapists' intentions about treatment decisions.

COMMENTS TO THE AUTHORS

13. Page 17, line 35: Please change "chose to refer" to "reported that they intend to refer for imaging or electrotherapy."

AUTHORS' RESPONSE
We thank the reviewer for the feedback and have removed the sentence for simplicity and clarity.

(Page 21, 3rd paragraph)

> Variations in findings across these studies may be due to differences in study methods. In the qualitative study, physiotherapists were asked to discuss barriers to using the recommendations, with the language of the recommendations identified as one of the barriers[5]. In the Best-Worst Scaling study, physiotherapists rated recommendations based on their willingness to follow them, without any additional context[6]. Meanwhile, in the current study, physiotherapists were presented with hypothetical clinical scenarios requiring more complex decision-making.

COMMENTS TO THE AUTHORS
14. Page 17, line 38: Sentence at beginning of sixth paragraph. While this may be the case, this statement is not accompanied by any references. Recommend removing, as the current study cannot support this kind of interpretation.

AUTHORS' RESPONSE
We agree with the reviewers' comments on the paragraph, and we have now removed the sentence and modified the discussion section based on the previous comments.

COMMENTS TO THE AUTHORS
15. General comment on sixth paragraph: This paragraph is exclusively about behaviour. Recommend removing this interpretation.
The final two sentences in this paragraph can go into a more nuanced discussion about intention being a component of behaviour. To modify behaviour, it is important to consider contextual factors.

AUTHORS' RESPONSE
Thank you for the suggestion. We have now removed the interpretation about behaviour and modified the paragraph as below:

(Page 22, 1st paragraph)

> Our study revealed that while physiotherapists showed a high level of familiarity and agreement with the Choosing Wisely recommendations, the recommendations did not influence their intentions to refer for imaging or use electrotherapy for low back pain. A possible explanation for the lack of effect in this current study is that awareness and agreement with guidelines are not sufficient to alter clinician intentions. Since intentions are behaviour-specific and can vary depending on the action and environment, it's important to understand how these factors interact[7]. Clinicians'

> decisions are shaped by elements such as such as cognitive bias[8], patient expectations[9, 10], organisational culture[11], lack of time[12] and clinical judgement[4], highlighting the complexity of behaviour change and the need for strategies that address both intentions and the context in which care is provided. Hence, to effectively drive change, it's essential to assess both current behaviours and the intentions behind them while considering key contextual factors that influence decision-making.

REVIEWER 2

COMMENTS TO THE AUTHORS

Thank you for the opportunity to review this paper. This was a randomised survey design exploring the choosing wisely recommendations in their original form and in an optimised version. The study design is excellent and I applaud the authors using a randomised design in this form of work, which is further strengthened by the large sample. Below, I present some considerations for the authors.

AUTHORS' RESPONSE

We thank the reviewer for their positive comments.

COMMENTS TO THE AUTHORS

I am curious why the authors did not further characterize the sample with something like the PABS-PT (https://pubmed.ncbi.nlm.nih.gov/15737810/), which is common in this field of research. The survey appears to have asked for self-reported perceptions of knowledge on these topics, which may be influenced by treatment orientation (e.g., a biomedical orientation compared to a biopsychosocial one when both rate 'extremely knowledgeable' may look like a very different answer…?) the effect of BM/BPS orientation has been shown regarding decision-making https://pubmed.ncbi.nlm.nih.gov/32947498/ https://pubmed.ncbi.nlm.nih.gov/37506584/ and treatment confidence/utilisation https://pubmed.ncbi.nlm.nih.gov/38054520/)

AUTHORS' RESPONSE

We agree that biomedical orientation compared to a biopsychosocial orientation may shape how physiotherapists interpret and apply knowledge, including responses to Choosing Wisely recommendations. We did not assess this because we thought the survey was already long enough and did not want to negatively impact participation rates. We do however agree it would be important for future studies to consider, so have mentioned this in the future research section.

(Page 22, 3rd paragraph)

> Future research could also explore how physiotherapists' biomedical versus biopsychosocial orientations influence their decision-making and responses to Choosing Wisely recommendations. Understanding this could help improve guideline adherence, clinical outcomes, and the implementation of evidence-based practices to optimise patient care[13, 14]. Another avenue for future research is further optimising the

> Choosing Wisely recommendations to increase their impact. For recommendations targeting low back pain management, this could involve integrating information on red flags or clarifying imaging-related recommendations to improve decision-making and adherence to guidelines.

COMMENTS TO THE AUTHORS
This was a large, global sample. Given the heterogenous contexts physiotherapists work in globally, what influence might this have on the outcome compared to if this was explored say only in the US or similar?

AUTHORS' RESPONSE
We agree with the reviewer's comment that the diverse contexts in which physiotherapists work globally could influence the outcomes. We have now added this to the discussion section. Please see below:

(Page 20, 4th paragraph)

> An important consideration in interpreting these findings is the characteristics of the participants in our study. First, most participants had good experience managing patients with low back pain, with 43% of physiotherapists seeing 6-50 patients with low back pain in the past year and 52% seeing more than 50 in the past year. This experience may have meant physiotherapists had established routines in managing patients with low back pain and were less likely to change their practice based on Choosing Wisely recommendations[4]. In contrast, a sample of less experienced physiotherapists may have been more receptive to practice change after reading the recommendations[5]. Second, most participants were from the United States (31%), United Kingdom (29%), and Australia (24%), countries with vastly different health systems. These differences likely influence how physiotherapists interpret and apply practice recommendations, could have possibly reduced the impact of the standardised Choosing Wisely recommendations.

COMMENTS TO THE AUTHORS
Was the study and analysis pre-protocolised somewhere such as OSF?

AUTHORS' RESPONSE
We did not pre-register the study on a clinical trial registry because our study was hypothetical/scenario-based, hence does not count as a true clinical trial. While we acknowledge it may have been useful to have the protocol registered on OSF, we do not believe this has introduced any bias since our findings were negative and therefore, not selectively reported.

COMMENTS TO THE AUTHORS
Optimised recommendations: I am curious why the authors did not further 'optimise' this? Why not give the red flags and talk in absence of cluster (as done well in this paper when talking about the role of assessment in exercise-based practice:

https://pubmed.ncbi.nlm.nih.gov/34676659/) for imaging-related items? This is not clear in this paper alone and seems the authors should expand more on their decision-making around optimisation in this paper.

AUTHORS' RESPONSE
We thank the reviewer for their comment. We based the optimisation of the recommendations on our Best-Worst study, which identified four characteristics (level of detail, qualified vs. unqualified language, positive vs. negative framing, and alternatives to low-value care) that could affect physiotherapists' willingness to follow the Choosing Wisely recommendations. We have added your suggestions of integrating red flags and addressing imaging-related items in our future research section.

(Page 22, 3rd paragraph)

> Future research could also explore how biomedical versus biopsychosocial orientations influence physiotherapists' decision-making and response to Choosing Wisely recommendations. Understanding this could help improve guideline adherence, clinical outcomes, and the implementation of evidence-based practices to optimise patient care[13, 14]. Another avenue for future research is further optimising the Choosing Wisely recommendations to increase their impact. For recommendations targeting low back pain management, this could involve integrating information on red flags or clarifying imaging-related recommendations to improve decision-making and adherence to guidelines.

COMMENTS TO THE AUTHORS
28% of the sample had a special interest in oncology? Did the authors specify this was related to non-cancer pain? Could this bias the result in this sample as a previous history of cancer is considered a red flag?

AUTHORS' RESPONSE
Thank you for your comment. We would like to clarify that less than 6% of the sample, or 28 respondents, expressed a special interest in oncology, not 28%. Out of these 28 respondents, only 19 (out of 275 total participants) attempted the third vignette question on the possible red flag. We do acknowledge that physiotherapists with a special interest in oncology may have been more adept at identifying red flags in vignette 3 and recommending imaging. However, given the small number of individuals with an oncology interest, we do not believe this would have introduced significant bias in the results.

COMMENTS TO THE AUTHORS
Discussion: the discussion is generally well written. I do feel there is a bit of a need for the reader to go and read the best-worst scaling study and qualitative study to completely grasp the authors points. As these (especially the best-worst scaling) appear to be quite linked to this study, I would suggest the authors provide some more key information to the reader.

AUTHORS' RESPONSE

We agree with the reviewer's comments regarding adding more information about the best-worst scaling study. These have been duly included in the discussion section of our manuscript. Please see below:

(Page 21, 2nd paragraph)

The findings of our study suggest that while physiotherapists generally support the Choosing Wisely recommendations, merely presenting the recommendations to physiotherapists, even with modified language to be clearer and more positive, is not sufficient to change physiotherapists' intentions about treatment decisions. Many physiotherapists in our study reported that the Choosing Wisely recommendations influenced their decision-making in the vignettes and indicated high familiarity and agreement with them. However, when they were presented with both original and modified versions of two APA Choosing Wisely recommendations targeting low back pain, no significant change was observed in their intentions to refer for imaging or use electrotherapy. This contrasts with our previous qualitative study (n=19 physiotherapists) which explored attitudes, views, and beliefs towards the APA original Choosing Wisely recommendations. This study identified that the language used in the recommendations was considered a barrier to their use[5]. Similarly, our best-Worst Scaling study involving 215 physiotherapists, assessed the effect of language on physiotherapists' willingness to follow the APA Choosing Wisely recommendations. We modified the six original recommendations based on four language characteristics (level of detail, strength- qualitied/unqualified, framing and alternatives to low-value care) to create 60 recommendations[6]. The study found that modifications to the language of the recommendations, such as providing more detail, offering alternatives to low-value care, and positively framing the recommendations, increased physiotherapists' willingness to adopt them[6].

References:

1. Qualtrics. ESOMAR 28. 28 questions to help research buyers of online samples. Updated 20/06/2014 [cited June 3 2024]. Available from: https://success.qualtrics.com/rs/qualtrics/images/ESOMAR%2028%202014.pdf.
2. Coombs DM, Machado GC, Richards B, Needs C, Buchbinder R, Harris IA, et al. Effectiveness of a multifaceted intervention to improve emergency department care of low back pain: a stepped-wedge, cluster-randomised trial. BMJ Quality & Safety. 2021;30(10):825-35.
3. Altinger G, Sharma S, Maher CG, Cullen L, McCaffery K, Linder JA, et al. Behavioural 'nudging' interventions to reduce low-value care for low back pain in the emergency department (NUDG-ED): protocol for a 2×2 factorial, before-after, cluster randomised trial. BMJ Open. 2024;14(3):e079870.
4. Zadro J, Peek AL, Dodd RH, McCaffery K, Maher C. Physiotherapists' views on the Australian Physiotherapy Association's Choosing Wisely recommendations: a content analysis. BMJ Open. 2019;9(9):e031360.
5. Kharel P, Zadro JR, Sundaram CS, McCaffery K, Dodd RH, McLennan C, Maher CG. Physiotherapists' attitudes, views, and beliefs about Choosing Wisely recommendations: A qualitative study. Musculoskelet Sci Pract. 2022;61:102610.

6. Kharel P, Zadro JR, Ferreira G, Howell M, Howard K, Wortley S, et al. Can language enhance physical therapists' willingness to follow Choosing Wisely recommendations? A best-worst scaling study. Braz J Phys Ther. 2023;27(4):100534.
7. Appleby B, Roskell C, Daly W. What are health professionals' intentions toward using research and products of research in clinical practice? A systematic review and narrative synthesis. Nurs Open. 2016;3(3):125-39.
8. O'Sullivan ED, Schofield SJ. Cognitive bias in clinical medicine. J R Coll Physicians Edinb. 2018;48(3):225-32.
9. Bishop TF, Cea M, Miranda Y, Kim R, Lash-Dardia M, Lee JI, et al. Academic physicians' views on low-value services and the choosing wisely campaign: A qualitative study. Healthc (Amst). 2017;5(1-2):17-22.
10. Ackerman SL, Gonzales R, Stahl MS, Metlay JP. One size does not fit all: evaluating an intervention to reduce antibiotic prescribing for acute bronchitis. BMC Health Services Research. 2013;13(1):462.
11. Van Dulmen SA, Verkerk EW, Born K, Gupta R, Westert GP, Kool RB. Challenges and Opportunities for Reducing Low-Value Care; A Response to Recent Commentaries. Int J Health Policy Manag. 2023;12:7954.
12. Kharel P, Zadro JR, Chen Z, Himbury MA, Traeger AC, Linklater J, Maher CG. Awareness and use of five imaging decision rules for musculoskeletal injuries: a systematic review. Int J Emerg Med. 2023;16(1):85.
13. Gibbs MT, Morrison NMV, Marshall PWM. Biomedical Beliefs Explain the Clinical Decisions Made by Exercise-Based Practitioners for People With Chronic Low Back Pain. Spine (Phila Pa 1976). 2021;46(2):114-21.
14. Gibbs MT, Last T, Marshall P, Jones MD. Are the attitudes and beliefs of Australian exercise-based practitioners associated with their use of, and confidence in, treatment modalities for people with chronic low back pain? Musculoskeletal Care. 2023.

VERSION 2 - REVIEW

**Reviewer**	**2**
**Name**	**Gibbs, Mitchell T.**
**Affiliation**	**University of New South Wales**
**Date**	**22-Apr-2025**
**COI**	

The authors have satisfied all revisions. I congratulate the authors on an important paper.
